# Variability in *SOD1*-associated amyotrophic lateral sclerosis: geographic patterns, clinical heterogeneity, molecular alterations, and therapeutic implications

**DOI:** 10.1186/s40035-024-00416-x

**Published:** 2024-05-29

**Authors:** Miaodan Huang, Yong U. Liu, Xiaoli Yao, Dajiang Qin, Huanxing Su

**Affiliations:** 1 State Key Laboratory of Quality Research in Chinese Medicine, Institute of Chinese Medical Sciences, Department of Pharmaceutical Sciences, Faculty of Health Sciences, University of Macau, Macao, China; 2 Laboratory for Neuroimmunology in Health and Diseases, Guangzhou First People’s Hospital School of Medicine, South China University of Technology, Guangzhou, China; 3 Department of Neurology, The First Affiliated Hospital, Sun Yat-Sen University, Guangdong Provincial Key Laboratory of Diagnosis and Treatment of Major Neurological Diseases, National Key Clinical Department and Key Discipline of Neurology, Guangzhou, China; 4 Key Laboratory of Biological Targeting Diagnosis, Therapy and Rehabilitation of Guangdong Higher Education Institutes, The Fifth Affiliated Hospital of Guangzhou Medical University, Guangzhou, 510799 China

**Keywords:** Amyotrophic lateral sclerosis, *SOD1* variants, Genotype, Geographic pattern, Genetic therapy

## Abstract

Amyotrophic lateral sclerosis (ALS) is a fatal neurodegenerative disease characterized by progressive loss of motor neurons, resulting in global health burden and limited post-diagnosis life expectancy. Although primarily sporadic, familial ALS (fALS) cases suggest a genetic basis. This review focuses on *SOD1*, the first gene found to be associated with fALS, which has been more recently confirmed by genome sequencing. While informative, databases such as ALSoD and STRENGTH exhibit regional biases. Through a systematic global examination of *SOD1* mutations from 1993 to 2023, we found different geographic distributions and clinical presentations. Even though different *SOD1* variants are expressed at different protein levels and have different half-lives and dismutase activities, these alterations lead to loss of function that is not consistently correlated with disease severity. Gain of function of toxic aggregates of SOD1 resulting from mutated *SOD1* has emerged as one of the key contributors to ALS. Therapeutic interventions specifically targeting toxic gain of function of mutant SOD1, including RNA interference and antibodies, show promise, but a cure remains elusive. This review provides a comprehensive perspective on *SOD1*-associated ALS and describes molecular features and the complex genetic landscape of SOD1, highlighting its importance in determining diverse clinical manifestations observed in ALS patients and emphasizing the need for personalized therapeutic strategies.

**Supplementary Information:**

The online version contains supplementary material available at 10.1186/s40035-024-00416-x.

## Background

Amyotrophic lateral sclerosis (ALS) is a devastating neurodegenerative disease characterized by progressive loss of motor neurons, leading to muscle wasting and, in general, a short life expectancy of 3 to 4 years after diagnosis as a result of respiratory failure [1]. The global burden of ALS is rising, and this trend is expected to continue in the coming decades [2]. Although ALS usually presents sporadically, approximately 5% to 10% of cases show familial clustering [3, 4], suggesting a genetic basis for ALS. This has spurred research into the genetic causes of this disease. Over the years, extensive genetic research has identified numerous genes associated with ALS, among which the most common ones are *SOD1* [5], *TARDBP* [6, 7], *C9orf72* [8, 9] and *FUS* [10]. Collectively, these four genes account for 60% of familial ALS (fALS) cases and 11% of sporadic ALS (sALS) cases, with *SOD1* alone accounting for 12% and 2% of fALS and sALS cases, respectively [11].

Initially identified by linkage analysis in fALS patients [12], the *SOD1* gene was subsequently confirmed as an ALS-associated gene in 1993 and found to contain specific mutations in fALS patients [5]. As a result, *SOD1* became a preferred choice for genetic testing in ALS patients for the majority of clinicians (68.3%) [13]. Since then, more than a hundred mutations, primarily missense mutations, have been identified in the *SOD1* gene. Despite the exponential accumulation of sequencing data with the advent of high-throughput genome sequencing, *SOD1* has consistently maintained its statistical significance in ALS genetics, ranking among the top ALS-associated genes in large-scale genome-wide association studies [14–19].

The growing number of variants not only in *SOD1* but also in other emerging ALS-associated genes has led to the generation of several databases of ALS-associated genes, such as ALSoD [20–22], STRENGTH, MinE [23] and NINDS Repository ALS collections [24]. While these databases offer extensive information on ALS-related variants, each tends to focus primarily on specific regions or populations, potentially overlooking regionally prevalent variants. For instance, ALSoD was established and continually updated by a group of researchers mostly from King's College London, with a primary focus on European cohorts. A recent study based on a cohort from ALSoD aimed at the *SOD1*-mediated ALS phenotype showed a decoupling between age of symptom onset and disease duration [25]. The study further led to the development of a web-utility tool that provides immediate access to and analytical framework of the database [26]. However, a more detailed examination of the study and the web-utility tool revealed a lack of consideration for some regionally prevalent variants in this database, such as R116G (mostly in Germany) [27], L145S (mostly in Poland) [28, 29], and C112Y (mostly in Asia) [30–35].

To comprehensively elucidate the global distribution of *SOD1* variants and investigate distinctive clinical presentations among different *SOD1* mutations, we conducted a rigorous systematic review of the research literature on ALS cases with *SOD1* mutations from 1993 to 2023 and examined critical aspects of *SOD1*-associated ALS, including geographic distribution of variants, diverse clinical phenotypes, and demographic characteristics of affected cohorts. Notably, SOD1 was initially identified as a 153-amino-acid protein due to the post-translational cleavage of its start methionine. However, it is now recognized as a 154-amino-acid protein. This discrepancy has led to variations in nomenclature for its variants across different literature sources. To maintain consistency, we followed the standard nomenclature based on the SOD1 sequence from NCBI (Accession Number: NC_000021), using A5V for A4V, D91A for D90A, G94A for G93A, and so forth. The mutational landscape of ALS spans the entire 154-amino-acid SOD1 protein and extends into the non-coding regions of the gene [36]. To further explore the underlying pathological mechanisms of these variants, we thoroughly reviewed the half-lives, protein levels, and activities of this protein from the literature and found that these parameters did not consistently correlate with disease severity. This review summarizes clinical features of *SOD1*-related ALS and the corresponding molecular alterations of variants, and discussses current and future therapeutic approaches that may provide a comprehensive perspective on the disease.

## Geographic distribution of *SOD1* variants

### Global discrepancies in variant reporting

Genetic studies have been conducted worldwide on large cohorts of patients with ALS (Additional file 1: Table S1). Initially, variants were identified through Sanger sequencing, covering all five exons of the *SOD1* gene, as well as flanking intronic sequences. Over the past decade, the advent of custom panels designed for next-generation sequencing has transformed the genetic screening landscape. Concurrently, comprehensive screening methods, including whole-exome sequencing, have emerged, resulting in significant advances in genetic analysis methods.

The inclusion of large cohorts, comprising both patients and controls, has facilitated the identification of pathogenic mutations and benign variations [37]. This holistic approach increases the robustness of genetic studies, contributing to a more comprehensive understanding of the genetic landscape of ALS.

**Fig. 1:**
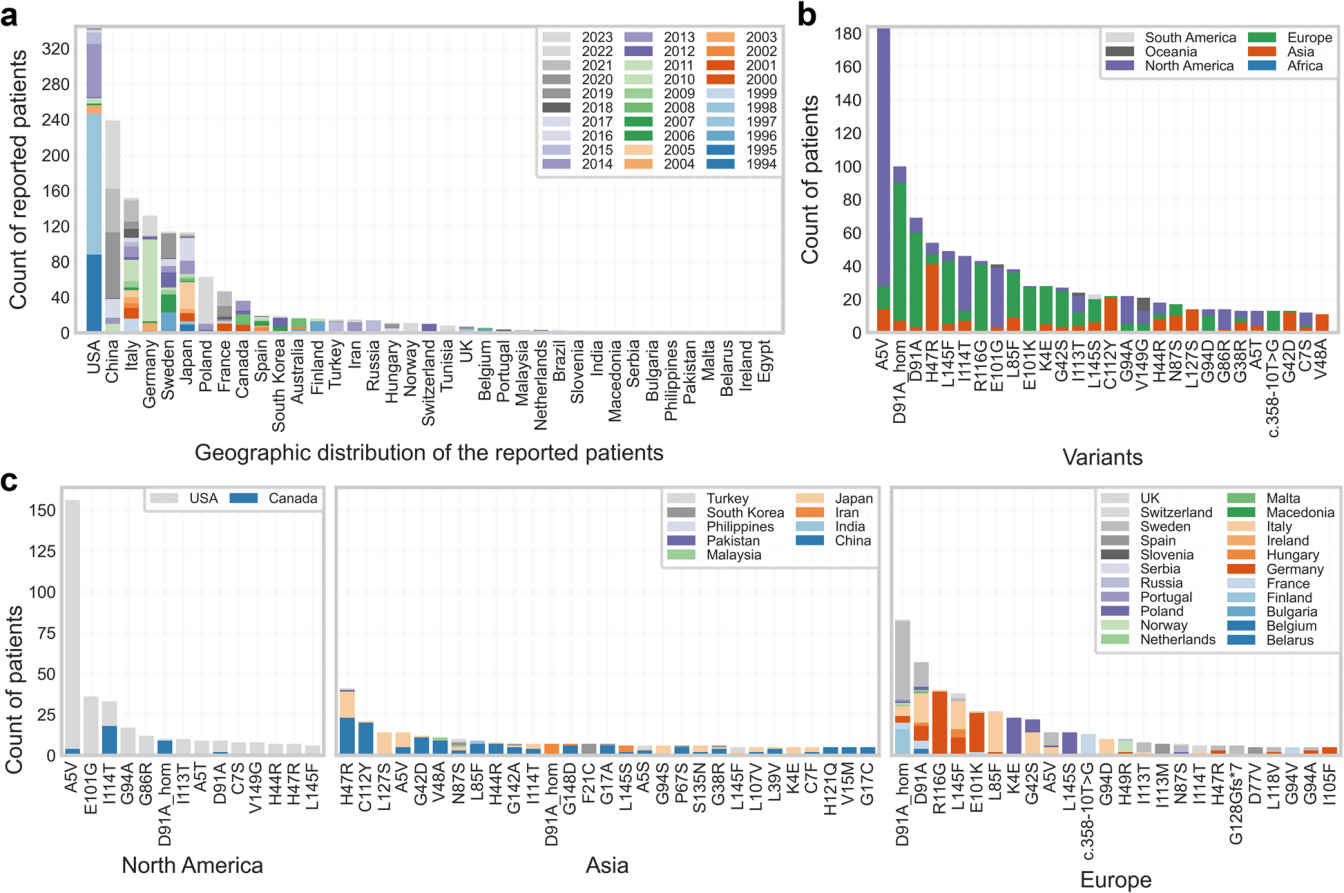
Geographic distribution of ALS patients with different *SOD1* variants. **a** Distribution of reported patients with *SOD1* variants (count > 10) across countries (1993 to 2023). PubMed was searched on June 18, 2023, with keywords such as SOD1, ALS, patient, variant, mutation, and humans, resulting in 901 publications. A total of 251 publications containing valid patient information were selected for data extraction. **b** Distribution of reported patients with different SOD1 variants (count > 10) across continents. **c** Distribution of reported patients with different SOD1 variants (count > 5) across countries. There were 8 cases of patients reported as SOD1 V149G carriers in Australia of Oceania which was not ploted in this panel. Variants in non-coding regions were expressed by denoting the nucleic acid site of variants with the symbol 'c.', according to the SOD1 gene sequence from the NCBI database (Accession Number: NC_000021)

It has been reported that the genetic architecture of ALS shows distinct patterns among populations [38]. In our study of reported cases of *SOD1*-associated ALS (Fig. 1a), the United States had prominent contributions to case reporting, particularly in the early stages of research on *SOD1* mutations associated with ALS, exhibiting frequencies of 12% to 49.1% in fALS and 0.5% to 1.5% in sALS [14, 15, 39].

China ranks second in the number of reported cases (Fig. 1a), showing a notable surge over the past decade from diverse medical centers across the country, with varied frequencies, ranging from 6.7% to 66.7% in fALS and 0% to 2.75% in sALS [30–32, 46–51]. In Europe, the majority of cases were reported during the second decade following the discovery of ALS-associated *SOD1* mutations, with frequencies ranging from 9.5% to 44% in fALS and 0% to 3.3% in sALS [28, 40–45].

The varied frequencies of *SOD1*-associated ALS cases reported within populations are intricately influenced by a multifactorial interplay involving genetic, environmental, and demographic determinants. Genetic heterogeneity, distinct ethnic genetic backgrounds, and regional discrepancies collectively contribute to variations in the prevalence of specific *SOD1* mutations associated with ALS. Environmental factors and lifestyle nuances across geographical regions further exert their impact on disease occurrence. Discrepancies in diagnostic methods, accessibility to healthcare services, and awareness of ALS introduce potential biases into case identification and reporting. Moreover, the dynamic evolution of genetic testing technologies and improved awareness over temporal epochs may contribute to temporal shifts in reported frequencies. Notably, the global inconsistencies in case reporting can, in part, be attributed to variations in regional developmental levels. The proportion of *SOD1* mutations within the broader genetic landscape shows substantial variability in fALS; however, it consistently maintains a prevalence around 2% in sALS across most regions.

### Geographical distribution spectrum and founder effect

To delve deeper into the geographical distribution patterns of various *SOD1* variants, we conducted an extensive survey of ALS cases with *SOD1* mutations across 37 countries spanning six continents (Fig. 1a, b). The origin of patients was defined as the country of the diagnostic institute unless otherwise specified. Our investigation revealed a diverse geographical distribution spectrum among different SOD1 variants (Fig. 1b, c), with certain variants exhibiting higher prevalence in specific regions. These findings offer insights into the evolutionary history of individual SOD1 variants.

The A5V variant emerged as the most prevalent variant globally, with a dominance in North America (Fig. 1b). Intriguingly, the prevalence of A5V was recognized early, only a year after identification of *SOD1* as an ALS-associated gene by the same research group [52]. Broom et al. estimated, by founder analysis, that the A5V mutation occurred ~ 12,000 years ago and further proposed that the mutation originated in Asia as the conserved minimal haplotype is more similar to the Asian than the European haplotype [53]. In addition, Saeed et al. conducted a thorough investigation [54] using high-throughput single-nucleotide polymorphism (SNP) genotyping on cohorts of ALS patients carrying the A5V mutation from North America and Europe and found that the A5V variant in North America could be traced back to two founders, one Amerindian and the other European, approximately 400 to 500 years ago.

Following A5V, the homozygous and heterozygous D91A variants claimed the second and third positions, respectively. Notably, D91A showed significant prevalence in Europe, with homozygous cases frequently observed in Scandinavia (a subregion in Northern Europe, including Denmark, Norway, and Sweden). Parton et al. traced the D91A variant using linkage disequilibrium and haplotype analysis [55, 56], and concluded that both homozygotes and heterozygotes descended from a single ancient founder. Their research suggested that the D91A mutation originated 22,000 years ago, with the homozygous allele established 3500 years ago. This allele was carried within the founding populations of Finland, expanding over the last 2000 to 3000 years.

In 1996, Hayward et al. performed haplotype analysis using the DISMULT program [57] to investigate the genetic background of six Scottish ALS patients carrying the I114T mutation. Their study revealed a shared ancestry of these patients, compared to a control group of 60 Scottish individuals, suggesting that the initial mutational event occurred over 10 generations ago.

The R116G emerged as a markedly regional variant, with a striking 90.7% of cases reported in Germany (calculated by our collection, data not shown). This variant is prominent within the German population, with haplotype analysis of four patients versus 90 controls suggesting a shared founding origin [27].

Similarly, the L85F represents another highly regional variant, with 61.5% of cases reported in Italy (calculated by our collection). Ceroni et al. proposed a shared ancestor among one sALS patient and three ALS families carrying the same L85F mutation in Italy [58]. It is worth noting that this conclusion was drawn based on the presence of the same mutation and regional ALS clustering, but it was not supported by haplotype analysis.

The notion of founder effects comes into play when a selected group of individuals, often originating from a different population or geographical area, carry a specific genetic mutation that becomes more widespread in subsequent generations [59]. The recognition of founder effects in ALS-associated *SOD1* mutations sheds light on the complex interplay between genetics and geography, ultimately influencing the patterns of disease.

Although there is a scarcity of comprehensive haplotype analyses for some prevalent *SOD1* variants, many of them show a tendency toward regional clustering (Fig. 1b, c), hinting at a potential shared ancestor for these variants. The proposition of ancestral genetic founders for prevalent *SOD1* variants indicates a significant genetic influence. This observation emphasizes the role of historical genetic events and population migration in shaping the landscape of ALS-associated *SOD1* mutations.

## Clinical phenotypic heterogeneity

### Different penetrance of variants

**Fig. 2:**
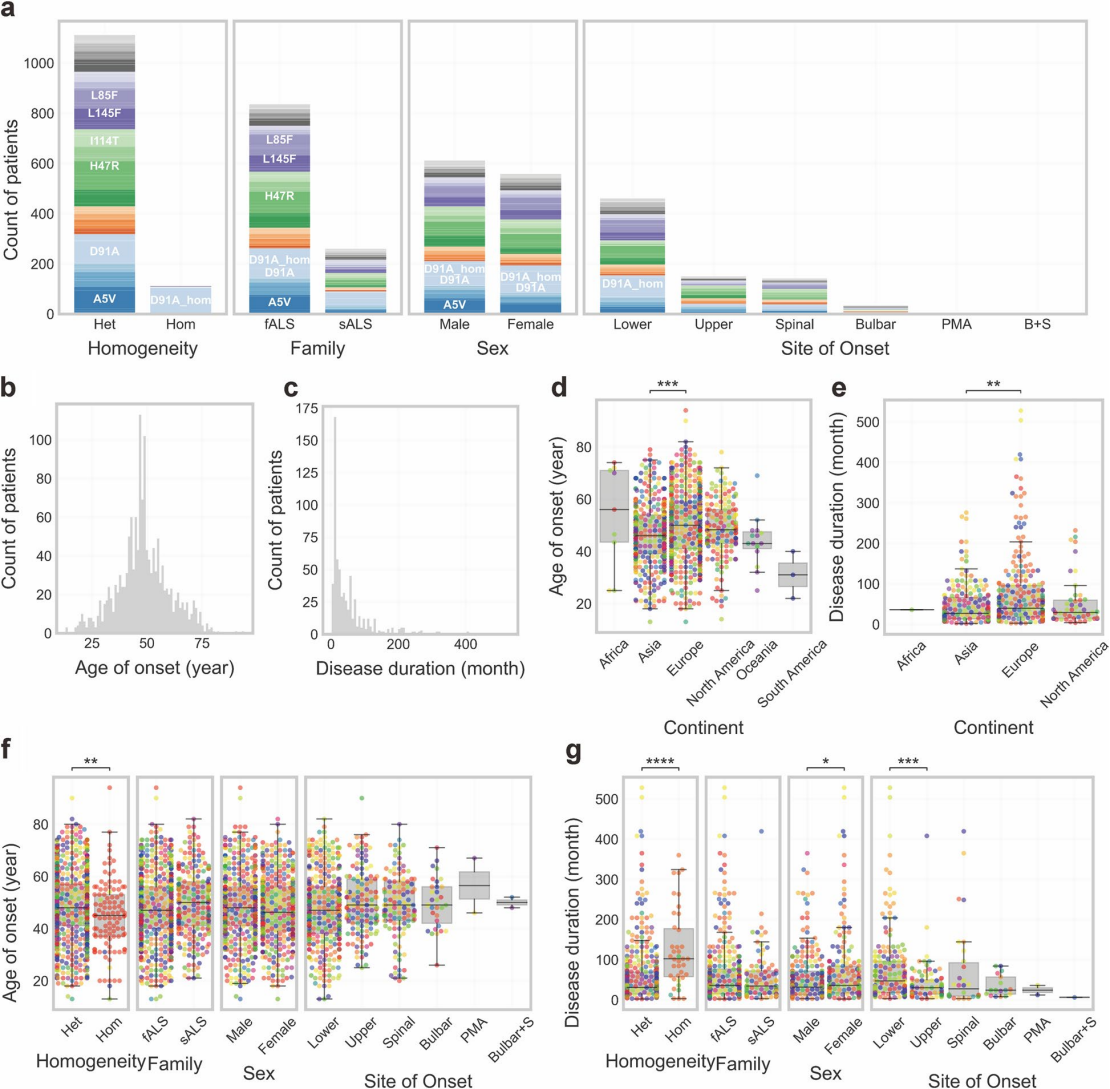
Clinical phenotypic heterogeneity. **a** Clinical phenotype distribution and reported patient counts by variants. **b** Distribution of age of onset in reported patients. **c** Distribution of disease duration in reported patients. Patients who were alive at the time of data collection in the original paper were excluded. **d** Age of onset in different continents. **e** Disease duration in different continents. **f** Age of onset distribution by clinical phenotype. **g** Disease duration by clinical phenotype. Swarmplots and boxplots (**d**-**g**) were generated using Matplotlib (version 3.5.2) and Seaborn (version 0.12.2). The Kruskal–Wallis test was used for overall statistical significance analysis across multiple groups and the Mann–Whitney test used for comparisons between two groups. Subsequently, *P*-values were adjusted with the Bonferroni correction to account for multiple comparisons. *P ≤ 0.05, **P ≤ 0.01, ***P ≤ 0.001, ****P ≤ 0.0001

Within the subgroup of ALS patients carrying *SOD1* mutations, there is a distinctive pattern of familial clustering, with approximately 76.3% of cases exhibiting familial ties (Fig. 2a). This finding stands in contrast to the broader population of ALS patients, where fALS comprises only 5%–10% of cases. This indicates a markedly higher tendency for familial clustering within the subgroup carrying *SOD1* mutations compared to the general prevalence of fALS in the broader ALS population. It should be noted that there are two types of fALS according to the revised El Escorial criteria [60], i.e., clinically definite and genetically determined fALS. In a previous survey, most clinicians agreed that patients who have a first-degree relative with ALS could be classified as fALS, and almost half of the respondents considered that being tested positive for a known ALS gene is sufficient for fALS diagnosis [13]. Patients with *SOD1* mutations are more likely to be classified as fALS under the latter criteria.

While *SOD1* is a prominent gene associated with ALS, its inheritance pattern is complex and does not strictly adhere to Mendel's laws. Some relatives of ALS patients carrying the same mutation show no disease symptoms. The phenomenon of incomplete penetrance occurs in different variants, as observed in elder carriers. For instance, an 80-year-old female, who had an affected daughter and was a carrier of the G62R mutation, exhibited reduced motor neuron function, but did not meet the diagnostic criteria for ALS [61]. In another case, the W33* mutation was found in a sALS patient, while the patient's mother, who was over 70 years old, remained asymptomatic [28]. Two N140H carriers in a Spanish family were ALS patients, while a 70-year-old woman carrying the same mutation was not affected [62]. Also, a G94D carrier died at 80 without any sign of ALS [63].

The phenomenon of incomplete penetration observed in ALS may arise from the diverse pathogenic potential of variants, underscoring the complex nature of ALS genetics. An algorithm was used to calculate variant penetrance from family history of disease and average family size in population-scale data [64]. This complexity has led to the formulation of a series of hypotheses aimed at explaining such cases, especially those involving variants with a larger patient pool.

Notably, some of the asymptomatic individuals can be classified as "presymptomatic carriers" as they have not yet reached the age of disease onset. Despite the absence of evident ALS symptoms, studies have revealed potential underlying abnormalities, such as metabolic alterations, among these carriers [65]. Notable findings indicate that the global DNA methylation levels in blood DNA of ALS patients are markedly higher compared to those in asymptomatic or mildly symptomatic carriers, as well as family members lacking *SOD1* mutations [66]. Moreover, a positive correlation has been found between global DNA methylation level and the duration of the disease in months [67]. Felbecker et al. proposed that some *SOD1* mutations may be part of an oligo- or epigenetic pattern of inheritance [68].

Most of the variants follow a dominant inheritance pattern as heterozygotes, with the exception of a few variants, primarily D91A, which exhibit partly homozygosity and a recessive inheritance pattern (Fig. 2a). The occurrence of D91A homozygotes [69] may be explained by the *cis*-acting regulatory polymorphism in the recessive haplotype proposed by Parton et al. [55]. Their study identified two distinct haplotypes for D91A, the recessive haplotype and the dominant haplotype. Compared to the dominant haplotype, the recessive haplotype harbors a mutation in a small non-coding region across *SOD1*. Remarkably, ALS did not develop among 139 D91A heterozygote individuals who had the recessive haplotype and had reached the age of risk for developing ALS, suggesting a potential protective effect of this additional mutation in non-coding regions. In addition, there are no differences in the activity or protein level of SOD1 in erythrocytes between ALS patients heterozygous for D91A with a dominant haplotype and unaffected individuals heterozygous for D91A with a recessive haplotype [70]. This initially suggested *cis*-acting regulatory protective hypothesis was refuted by Sanger sequencing of PCR amplicons covering the entire 115-kb conserved *SOD1* D91A haplotypic region, where SNP analysis did not support the presence of a genetic modifying factor with a putative neuroprotective effect [71]. Despite these findings, the precise mechanisms underlying the coexistence of dominant and recessive inheritance patterns for D91A remain elusive.

In a study of a Canadian family with the G28delGP mutation, alternative splicing of *SOD1* was found to influence disease severity and phenotypic variability [72]. Quantitative real-time polymerase chain reaction analysis on the white blood cells from eight family members with different mutation status revealed three types of *SOD1* transcript: full-length *SOD1* transcript, transcript lacking exon 2, and *SOD1* lacking both exons 2 and 3. The deletion of codons 28 and 29 eliminates an exonic enhancer sequence for the splicing factor SC35, leading to poor recognition of exon 2 by the splicing machinery, resulting in the exclusion of exon 2 from *SOD1* mRNA, thus decreasing allelic expression. In a homozygous G28delGP patient, the full-length isoform was decreased, while the exon 2-lacking isoform was increased compared to the heterozygous and wild-type family members. This pattern was also observed in the human spinal cord, except for the isoform lacking both exons 2 and 3.

These studies provide valuable insights into the genetics of ALS. The elevated DNA methylation levels and the impact of alternative splicing on gene expression all point to the complex interplay of genetic, epigenetic, and transcriptional factors influencing the progression of ALS. Collectively, these findings offer promising avenues for future research, sparking optimism for enhanced comprehension, treatment, and potential prevention of ALS.

### Disease severity

The age of onset and disease duration are key indicators of disease severity among *SOD1*-related ALS patients. Across the entire cohort in our collection, the age of onset exhibits a Gaussian distribution pattern (Fig. 2b), with an average age of 48.53 years (*n* = 1436, Additional file 1: Table S2). In contrast, the disease duration exhibits a positively skewed distribution (Fig. 2c), with a mean of 56.17 months and a median of 29.00 months (*n* = 636, Additional file 1: Table S3), highlighting the significant variability in disease progression within this population. Notably, when including the patients who were still alive at the time of recording, the diease duration extends to a mean of 63.64 months and a median of 38.00 months (*n* = 1239). One factor for the extension could be advancements in medical interventions and therapies that have increased the life span of ALS patients. Additionally, the inclusion of patients still alive at the time of recording introduces a bias towards longer durations, as those individuals have not yet reached the endpoint of their disease progression (death is considered as the endpoint in our collection).

We have identified a phenomenon of decoupling between the age of onset and disease duration, mirroring the findings reported by Opie-Martin et al*.* [25]. A more detailed analysis of the data revealed that the disease duration may indeed show correlations with the age of onset in specific variants (Table 1).

Although different variants exhibit similar distributions in terms of age of onset, those with longer life expectancies tend to display discrete distributions of disease duration, as evidenced by high variances in Table 1. To evaluate the potential relationship between age of onset and disease duration, we conducted linear regression analyses on these two factors. Taking the D91A variant as an example, the analysis revealed a statistically significant relationship, with* P*-values consistently below 0.01, despite the modest *R* values (Table 1). Linear models for both heterogeneous and homogeneous D91A consistently showed a negative trend, indicating that an earlier age of onset is associated with an extended disease duration among individuals with this variant. This pattern extends to the L145F and N87S variants. Notably, the linear model for the N87S variant has a robust fit, characterized by an exceptionally low* P*-value of < 0.0001 and an impressive *R* value of -0.93 (*n* = 8).

**Table 1:** Linear regression analysis for age of onset and disease duration (data from deceased patients,* n* > 5)

**Variant**	***n***	**Linear regression**			**Age of onset**			**Disease duration**		
		**Formula**	***R***	***P* value**	**Mean (years)**	**variance**	**skewness**	**Mean (months)**	**variance**	**skewness**
**Total**	490	y = –1.01x + 112.03	–0.18	0.00005	49.22 ± 13.73	188.55	0.04	56.17 ± 76.15	5799.42	2.70
**A5V**	36	y = 0.02x + 13.78	0.03	0.85311	48.12 ± 13.08	170.98	–0.57	12.90 ± 7.80	60.77	0.96
**D91A_hom**^**a**^	34	y = –3.41x + 308.60	–0.53	0.00132	50.34 ± 15.08	227.54	0.53	136.86 ± 97.38	9482.72	0.74
**I114T**	21	y = –1.54x + 131.21	–0.41	0.06456	57.33 ± 9.39	88.19	–0.80	45.10 ± 35.28	1244.59	1.18
**D91A**	19	y = –3.38x + 300.71	–0.58	0.00863	51.73 ± 13.86	192.05	0.47	125.71 ± 80.26	6441.54	0.34
**L145F**	19	y = –6.40x + 450.66	–0.64	0.00317	49.80 ± 15.57	242.52	–0.27	130.81 ± 155.84	24287.06	1.78
**G42S**	17	y = –0.10x + 17.39	–0.25	0.32762	47.82 ± 11.01	121.20	–0.80	12.71 ± 4.27	18.21	–0.13
**L85F**	16	y = 0.77x + 23.03	0.30	0.25493	41.50 ± 11.96	143.12	–0.27	55.12 ± 30.59	935.98	1.28
**c.358–10T>G**	11	y = 0.38x + 10.31	0.23	0.49288	62.09 ± 12.71	161.54	0.76	34.00 ± 20.92	437.82	0.77
**C112Y**	10	y = 1.29x – 16.29	0.73	0.01711	48.95 ± 11.94	142.53	0.24	46.77 ± 21.14	447.02	0.74
**H47R**	8	y = –4.78x + 427.05	–0.57	0.13950	46.50 ± 8.48	71.86	–0.26	201.00 ± 70.96	5034.75	–0.68
**K4E**	8	y = 1.88x – 28.51	0.29	0.48474	58.38 ± 8.20	67.23	0.37	81.00 ± 52.90	2798.25	0.53
**I113T**	8	y = 0.03x + 20.59	0.02	0.96751	64.88 ± 8.10	65.61	–0.55	22.62 ± 14.63	213.92	1.59
**N87S**	8	y = –1.65x + 156.15	–0.93	0.00079	57.62 ± 17.80	316.73	–0.35	61.12 ± 31.53	994.36	0.37
**C7S**	8	y = –3.44x + 262.09	–0.69	0.05687	51.12 ± 10.06	101.11	0.35	86.00 ± 50.00	2500.00	0.61
**H44R**	7	y = 0.91x – 32.81	0.83	0.02214	50.55 ± 8.67	75.21	1.05	15.40 ± 9.58	91.72	1.56
**R116G**	6	y = –0.60x + 52.51	–0.75	0.08710	55.17 ± 11.87	140.81	–0.51	19.45 ± 9.51	90.35	0.52
**L127S**	6	y = –1.66x + 168.62	–0.74	0.09561	52.50 ± 15.48	239.58	–0.05	81.33 ± 34.98	1223.89	–0.22
**G94S**	6	y = 2.22x – 25.25	0.68	0.14102	53.33 ± 12.76	162.89	–0.82	93.00 ± 41.90	1756.00	0.12
**G148D**	6	y = 0.13x + 7.76	0.26	0.61584	44.83 ± 10.02	100.47	0.50	13.80 ± 5.15	26.53	0.96
G128Gfs*7	6	y = 0.73x – 26.10	0.67	0.14906	63.00 ± 8.62	74.33	–0.17	19.67 ± 9.41	88.56	–0.40
**S135N**	6	y = 0.34x + 19.97	0.11	0.83014	57.13 ± 14.67	215.09	0.47	39.17 ± 43.33	1877.81	1.35
**L107V**	6	y = 1.51x – 35.45	0.46	0.35536	39.14 ± 7.13	50.81	–0.22	24.40 ± 23.20	538.42	1.50

^a^As most *SOD1*-related ALS patients are heterozygotes, ALS patients with *SOD1* homozygotes are designated as "variant_hom"

Some variants, specifically A5V, G42S, H44R, R116G, and G128Gfs*7, exhibit a more centralized pattern of disease duration, characterized by low variance and reduced life expectancy, indicating that they are aggressive yet relatively uniform variants.

As the most prevalent genetic variant, A5V is associated with a remarkably shortened life expectancy (12.90 ± 7.80 months, *n* = 36). This finding is consistent with the results of a retrospective cohort study conducted across 15 medical centers in North America, which examined the medical records of 175 ALS patients with genetically confirmed *SOD1* mutations [73].

To explore additional factors contributing to disease severity, a significance test was performed. Geographically, the age of onset in Asia was 46.66 ± 12.16 years, significantly younger than that in Europe, where it averaged 49.99 ± 13.51 years (*P* = 0.000293, Fig. 2d). The median disease duration in Asia (27 months) was notably shorter compared to that in Europe (40 months) (*P* = 0.000293, Fig. 2e). The younger age of onset and shorter median disease duration in the Asian population may have a genetic or environmental basis since it could not be simply explained by regional healthy life expectancy, which is 60.7–71.2 years in Asia compared to 62.0–68.5 years in Europe accroding to a comprehensive demographic analysis for the Global Burden of Disease between 1950 and 2019 [74]. No significant differences were observed in disease severity between fALS and sALS (Fig. 2f, g). Surprisingly, homozygous cases exhibited a relatively milder disease severity, characterized by a younger age of onset but an extended disease duration compared to heterozygous cases (Fig. 2f, g).

These findings provide valuable insights into the diverse clinical presentations of *SOD1* variants and the multifactorial nature of ALS progression, emphasizing the need for a personalized approach for diagnosis and treatment of this disease based on the specific genetic and geographical context.

### Clinical onset pattern

The prevalence (95.07%) of spinal onset, mainly characterized by lower limb involvement (58.2%), in *SOD1*-related ALS cases, suggests this disorder as a spinal-onset disease (Fig. 2a). Conversely, bulbar onset, representing merely 4.4% of cases, is less frequent. This distribution reveals the primary anatomical localization of pathological processes associated with *SOD1* mutations within the spinal region. Autopsies of five ALS patients with A5V mutation indicated a predominant lower motor neuron involvement, and no involvement or mild involvement of upper motor neurons [75]. Remarkably, patients manifesting lower limb onset exhibit a more favourable prognosis, characterized by an extended life expectancy compared to those with upper limb onset (Fig. 2g). This highlights the potential clinical relevance of the initially affected areas in the neurodegenerative cascade associated with *SOD1* mutations.

The comprehensive analysis of the site of onset in *SOD1*-related ALS patients not only provides valuable insights into the clinical presentation of the disease, but also serves as a potential prognostic indicator. Further studies could delve into elucidating the underlying mechanisms and contributing factors for the observed differences in prognosis depending on the site of onset.

### Sex differences

Within the cohort of ALS patients with *SOD1* mutations, 52.31% (*n* = 612) are males and 47.69% (*n* = 558) are females (Fig. 2a). Although no significant difference was found in the age of onset between sexes, females had a longer life expectancy with a median of 36 months (*n* = 261), compared to their male counterparts with a median of 30 months (*n* = 209) (Fig. 2g). Notably, a study involving a Chinese cohort also reported longer survival among female *SOD1*-mutant ALS patients than males [48].

For the L127S mutation, a low penetrance was found in females. Consistently, in a case report, two Japanese male ALS patients in a pedigree both carried the L127S mutation, while their mothers, who were also carriers of the mutation, remained unaffected over 80 years old [76].

Male low-copy and high-copy human SOD1 G94A transgenic mice show significantly earlier age of onset than female transgenic mice [77]. The hSOD1G93 transgenic rat model of ALS shows sexual dimorphism in disease onset and progression [78]. In addition, the SOD1 concentration in the cerebrospinal fluid (CSF) is significantly higher in male ALS patients than in female ALS patients [79].

The better survival observed among female *SOD1*-mutant ALS patients may be attributed to a complex interplay of genetic, hormonal, and environmental factors. Further research involving larger and more diverse cohorts is necessary to elucidate the specific genetic and hormonal mechanisms contributing to the longer survival of female *SOD1*-mutant ALS patients.

## Molecular alterations of* SOD1* variants

The variants collected in our dataset include 2 silent mutations, 3 double-amino-acid mutations, 5 in-frame deletion mutations, 7 stop-gain mutations, 9 frame-shift mutations, and 164 single-amino-acid mutations. The remaining 11 variants have mutations in the non-coding regions including a point mutation in the TATA box, a single-nucleotide mutation in the 5’ untranslated region (UTR), a single-nucleotide mutation in the 3’ UTR, a nucleotide deletion and 7 single-nucleotide mutations in introns (Table 2).

**Table 2:** Genetic localization of variants

**Region**	**Type**	**Location**	**Variant**	**a.o. (years)**	**d.d. (months)**
**coding**	**AA deletion **Mean a.o.: 51.9, *n* = 7 Mean d.d.: 63.5, *n* = 4	**exon 1**	I19del	80.0, *n* = 1	na
		**exon 2**	G28delGP	51.0, *n* = 1	48.0, *n* = 1
		**exon 4**	S106delSL	52.0, *n* = 1	31.0, *n* = 1
		**exon 5**	G131delGNEE, E133del	45.0, *n* = 4	87.5, *n* = 2
	**AA point mutation** Mean a.o.: 48.4, *n* = 1355 Mean d.d.: 57.1, *n* = 587	**exon 1**	K4E, A5V, A5S, A5T, A5F, V6L, V6M, C7F, C7W, C7S, C7G, L9Q, L9V, G11V, G11A, G11R, D12Y, G13R, V15M, V15G, G17A, G17H, G17C, G17S, N20S, F21V, F21C, F21L, E22G, E22K, Q23L, Q23R	48.3, *n* = 319	25.8, *n* = 169
		**exon 2**	V32A, W33G, G38V, G38R, G38C, L39V, L39R, E41G, G42D, G42S, H44R, F46C, F46S, H47R, V48A, V48F, H49R, H49E, E50K	46.5, *n* = 173	69.4, *n* = 66
		**exon 3**	C58Y, G62R, N66T, N66S, P67S, P67R, P67A, P67T, P67L, L68P, S69P, R70G, H72Y, G73C, G73D, G73S, P75S, D77Y, D77V, R80S	49.7, *n* = 53	74.3, *n* = 26
		**exon 4**	H81Y, H81R, D84H, D84N, D84G, L85F, L85V, G86R, G86C, N87S, N87I, N87K, N87D, V88A, A90T, A90F, A90V, D91A, D91N, D93G, G94V, G94C, G94R, G94A, G94D, G94S, V95G, A96T, I100V, E101G, E101K, D102N, D102G, D102E, D102H, I105F, S106L, L107V, L107F, D110Y, C112Y, I113T, I113M, I113F, I114T, I114F, G115A, R116S, R116G, L118V, V119L, V119M	48.6, *n* = 610	71.9, *n* = 245
		**exon 5**	V120L, V120F, H121Q, H121R, E122G, D125V, D125G, D126N, L127S, G128V, G128R, E134V, E134*, E134G, E134K, S135N, S135T, T138R, T138A, G139V, G139E, N140D, N140K, N140H, G142A, G142E, L145S, L145F, A146T, C147R, G148D, G148N, G148S, G148C, V149G, I150T, I150V, I150M, I152T, I152S, A153P, Q154R	49.2, *n* = 200	62.2, *n* = 81
	**Double AA point mutation** Mean a.o.: 45.5, *n* = 4 Mean d.d.: 222.0, *n* = 2	**exon 3&4**	D91A&I114T, D91A&D97N	38.7, *n* = 3	222.0, *n* = 2
		**exon 4**	P67S&D91A	66.0, *n* = 1	na
	**Silent mutation** Mean a.o.: 67.3, *n* = 3 Mean d.d.: 62.5, *n* = 2	**exon 3**	S60S	77.0, *n* = 1	12.0, *n* = 1
		**exon 5**	A141A	62.5, *n* = 2	113, *n* = 1
	**Truncating mutation** Mean a.o.: 47.1, *n* = 37 Mean d.d.: 31.1, *n* = 21	**exon 2**	W33*	41.0, *n* = 1	na
		**exon 4**	K92Rfs*9, S108Lfs*15	66.0, *n* = 3	12.0, *n* = 1
		**exon 5**	D125Tfs*24, L127Gfs*6, L127*, G128Gfs*7, K129Kfs*4, G131Kfs*2, N132Qfs*5, E133*, E134*, K137*, N140*, G142*, L145Ffs*3	45.6, *n* = 33	32.1, *n* = 20
**non-coding**	**NC deletion** Mean a.o.: 64.0, *n* = 2 Mean d.d.: 51.0, *n* = 2	**intron 2**	c.169+50delAACAGTA	64.0, *n* = 2	51.0, *n* = 2
	**point mutation** Mean a.o.: 53.9, *n* = 28 Mean d.d.: 33.8, *n* = 18	**3'UTR**	c.*249T>C	46.0, *n* = 1	30.0, *n* = 1
		**5'UTR**	c.-46C>T	42.0, *n* = 1	66.0, *n* = 1
		**TATA**	c.-109A>G	56.0, *n* = 2	60.0, *n* = 1
		**intron 2**	c.170-20T>C, c.170-7T>C	64.0, *n* = 2	na
		**intron 3**	c.239+34A>C, c.240-3T>C, c.240-7T>G	42.1, *n* = 9	19.8, *n* = 4
		**intron 4**	c.358-10T>G, c.358-11A>G	61.6, *n* = 13	34.0, *n* = 11

a.o.: age of onset (years) d.d.: disease duration (months)

### Variants in non-coding regions

**Fig. 3:**
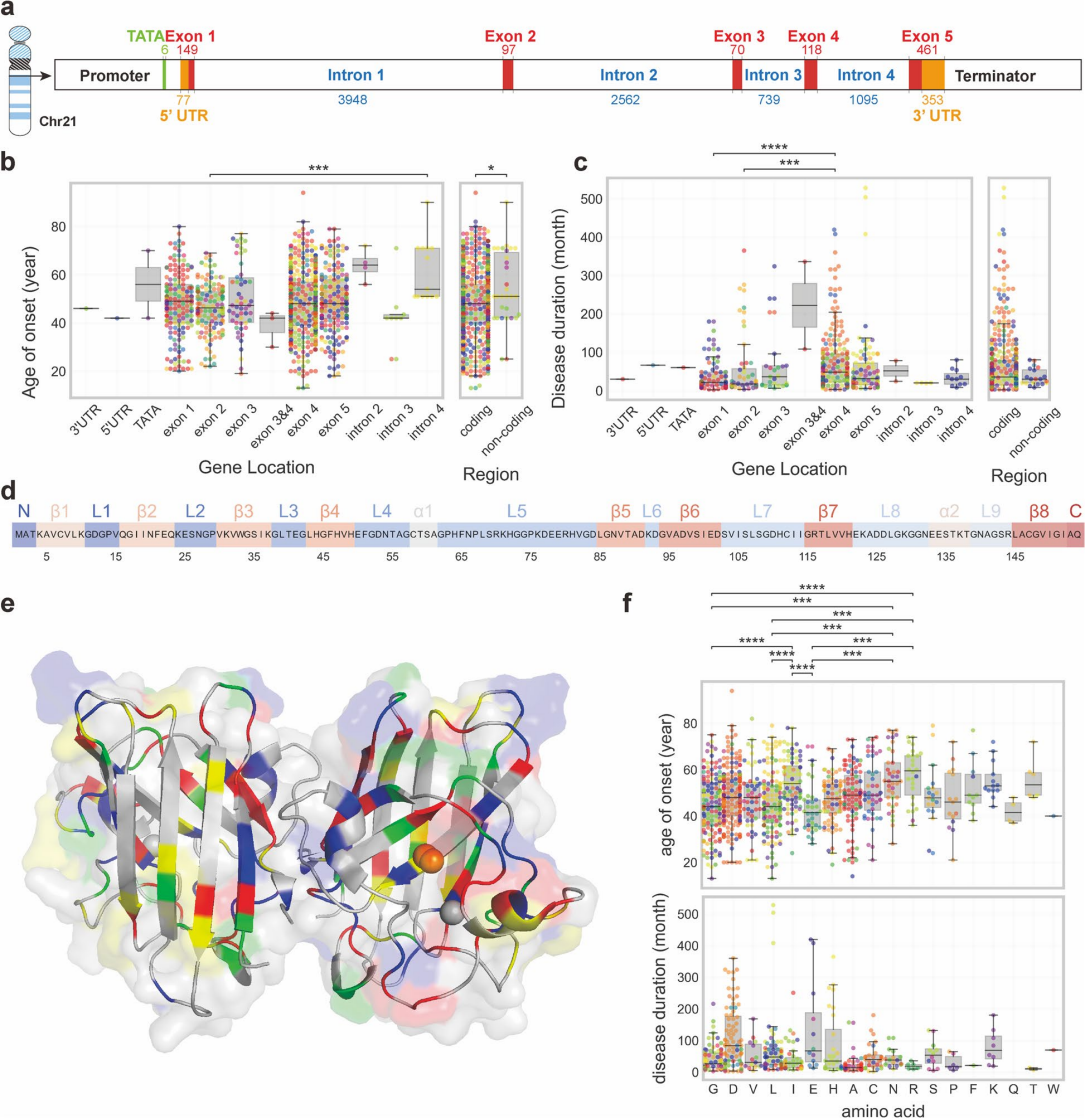
Molecular alterations of *SOD1* variants. **a** Location of *SOD1* gene (Accession No: NC_000021). **b** Age of onset distribution among patients with different genetic locations of *SOD1* variants. **c** Disease durations of patients with different genetic location of *SOD1* variants. **d** Secondary structure of SOD1 protein. The secondary structural elements, including alpha-helices and beta-strands, were identified using the PyMOL software (version 2.5.0) based on the 3D crystal structure of the human SOD1 protein (PDB id: 2V0A). **e** The 3D crystal structure of the human SOD1 protein from the Protein Data Bank (PDB id: 2V0A). Codon colors represent the associations of the variants with the mean disease duration and the mean age of onset. Green represents disease duration longer than 56.17 months (the mean disease duration of the total *SOD1*-related ALS patients) and the age of onset older than 48.51 years (the mean age of onset of the total *SOD1*-related ALS patients), blue indicates longer duration and younger age, yellow signifies shorter duration and older age, and red indicates shorter duration and younger age. (**f**) Age of onset and disease duration of patients with different amino acid sites of variant. Significance was analyzed using Kruskal–Wallis test and *P*-values were adjusted to account for multiple comparisons using Bonferroni correction. *P ≤ 0.05, ** *P* ≤ 0.01, *** *P* ≤ 0.001, ****P ≤ 0.0001

The *SOD1* gene is located on chromosome 21q22.1 and contains five exons with a genomic size of 9239 bp (Fig. 3a). Most variants are located within exons, particularly exons 4 and 5, correlating with earlier onset compared to those in non-coding regions. Variants within exon 2 are associated with a younger onset and shorter duration (Fig. 3b, c). Notably, exon 2 encodes β3, loop 3 and β4, which form the characteristic 'Greek key' structure in SOD1-like proteins [80, 81].

Intriguingly, several variants are located on the intronic regions, influencing crucial elements for RNA splicing. RNA splicing, orchestrated by the spliceosome, is a precise process involving the removal of introns and joining of exons from pre-mRNA to form mature mRNA [82–84]. Mutations in spliceosome-recognized sequences at intron–exon boundaries, such as the 5' splice site, branchpoint, polypyrimidine tract, and 3' splice site, lead to disordered splicing and amino acid sequence alterations. Notable examples include polypyrimidine tract mutations, such as c.170-20 T > C and c.170-7 T > C in intron 2, and c.240-3 T > C in intron 3, which have been identified in ALS patients. Additionally, mutations introducing new splicing sites, like c.240-7 T > G (E78insSI), c.358-10 T > G (V119insFLQ) [85], and c.358-11A > G (V119fs*6), result in distinct consequences. Another example is c.358-304C > G, which introduces a new donor splice site after a cryptic exon on intron 4, leading to a 43-bp pseudoexon and a full ALS penetration [86] (not included in our dataset since the patient information was not available).

A few mutations in UTRs have been identified, including c.-109A > G, c.-46C > T, and c.*249 T > C in Russian ALS patients [42], and c.-64C > T, c.-6G > T, and c.*248A > C in Polish ALS patients [28] (not included in our dataset since the patient information was not available). Specifically, the c.-109A > G mutation alters the *SOD1* TATA box from TATAAA to TGTAAA, resulting in decreased gene expression compared to the wild-type [87].

Some intronic mutations, like c.169 + 50delAACAGTA [88] and c.239 + 34A > C [88, 89], have been reported in ALS patients without further exploration. In addition, intrinsic alternative splicing of *SOD1* remains to be studied, with only two reported studies in the year 2000 [90, 91]. Mutations in UTRs can influence gene expression by potentially affecting transcription factor binding, leading to altered transcription levels [92]. Additionally, 3' UTR mutations may play a role in regulating mRNA translational efficiency, localization, and stability [93–96]. A more comprehensive exploration of these regions could reveal novel insights into the regulatory mechanisms and functional consequences of such variants, contributing to a holistic understanding of the genetic landscape associated with ALS and related disorders. The limited number of patients with intronic mutations poses a challenge in achieving sufficient statistical power to establish their pathogenicity.

### Variants in coding regions

SOD1 is a highly conserved protein of 154 amino acids that folds into an eight-stranded Greek Key β-barrel structure (Fig. 3d) [80, 81] and binds one atom of copper and one atom of zinc (Fig. 3e).

Most of the variants are missense mutations resulting from single point mutations. An important observation from our study is that a majority of patients have single point mutations involving substitution of residues such as aspartic acid (Asp, D), glycine (Gly, G), leucine (Leu, L), alanine (Ala, A), and isoleucine (Iso, I) (Fig. 3f).

The negatively charged aspartic acid has the potential to modify the charge distribution within the protein, thereby influencing interactions with other molecules or cellular structures. Glycine is unique due to its lack of side-chain carbons, which confers conformational flexibility of the protein structure. On the other hand, leucine, alanine, and isoleucine are hydrophobic amino acids, and alterations in these residues may affect the hydrophobic interactions crucial for protein folding and stability.

Despite these recurrent residues, specific residues involved in metal binding, disulfide-bond formation, and post-translational modifications (PTMs) are substituted in some ALS-related variants [97–102]. PTMs have been experimentally identified in various amino acids of SOD1, including tryptophan (Trp, W), lysine (Lys, K), and serine (Ser, S). Trp33 serves as both an oxidation site [103, 104] and a nitration site [105–107]. Variations such as W33G and W33* have been observed at this location. Lysine undergoes mutations (K4E, K129Kfs*4) and can be modified through glycation [108] and acetylation [109]. Ser60 functions as a phosphorylation site [110], and a synonymous mutation at this site has been detected in an Italian patient with sALS [40]. Although this isolated case is not conclusively significant as a pathological mutation, if proven otherwise, the pathogenicity may be caused by differential rates depending on specific codons [111] or codon bias [112].

Histidine residues (His, H) are involved in metal binding and serve as active sites in enzymes, playing a crucial role in SOD1 activities. Several ALS-related *SOD1* variants involve the replacement of copper-binding histidine residues (H47R, H49E, H49R, H121Q, and H121R) and zinc-binding histidine residues (H72Y, H81Y, and H81R).

In addition, cysteine residues (Cys, C) contribute significantly to SOD1 function. The thiol group of cysteine is highly reactive and facilitates the formation of disulfide bonds between two cysteine residues. Several ALS-related cysteine mutations (C7F, C7G, C7S, C7W, C58Y, C112Y, C147R) have been identified. Among them, C7 serves as the palmitoylation and nitration site [105], and a conserved intramolecular disulfide bridge forms between C58 and C147, which is crucial for SOD1 stability in the intracellular reducing environment [113, 114]. C112 is a multifunctional residue, serving as a hub of PTMs such as redox modification [115], palmitoylation, and nitration [105]. These modifications may impact the aggregation and cytotoxicity of mutant SOD1 molecules [116]. Moreover, abnormal binding of copper to the C112 has been suggested as a significant contributing factor to the altered behavior of mutant SOD1. This may explain the benefits of controlling copper access to mutant SOD1 in G94A transgenic mice [117].

The different properties of these amino acids, including charge, flexibility, and hydrophobicity, indicate that alterations in these residues might lead to changes in the overall protein structure. Therefore, understanding the specific consequences of these variations could provide important insights into the pathogenic mechanisms associated with SOD1 and its role in related disorders. The limited involvement of PTM target residues in ALS might be attributed to their functional significance.

Notably, truncating mutations by substitutions (c.98G > A for W33*, c.380 T > A for L127*,c.397G > T for E133*, c.409A > T for K137*, c.424G > T for G142*), insertions (c.320_321insT for S108Lfs*15, c.383_384insTGGG for G128Gfs*7, c.379_380insTGGGCAAAGG for N132Qfs*5, c.396_399dup for E134*, c.417_418insT for N140*, c.435GinsCGTTTA for L145Ffs*3), and deletions (c.275_276del for K92Rfs*9, c.379_380del for L127Gfs*6, c.387_388del for K129Kfs*4, c.389_390del for G131Kfs*2) of nucleotides generate premature termination codons. All premature termination codons originating from these truncating mutations can escape the nonsense-mediated mRNA decay because they are (1) in the last exon, (2) within 50–55 nucleotides upstream of the last exon-exon junction, or (3) within 150 nucleotides from the start codon [118, 119].

### Molecular features of variants

SOD1 functions as a key metalloenzyme, serving as an important reservoir for copper and zinc ions. As the primary intracellular superoxide dismutase, SOD1 catalyzes the dismutation of superoxide radicals (O^2−^) into molecular oxygen (O_2_) and hydrogen peroxide (H_2_O_2_). This enzymatic activity is critical for neutralizing deleterious reactive oxygen species (ROS), thereby serving as a crucial defense mechanism against oxidative stress. Thus, SOD1 plays a major role in maintaining cellular integrity and mitigating the detrimental effects of oxidative reactions. Besides the superoxide dismutase function, SOD1 has additional peroxidase activity and nitrosylation activity, contributing to its multifaceted roles in cellular homeostasis [113]. Remarkably, despite its well-established roles as an enzyme and a buffer of Cu and Zn, SOD1 may have additional intrinsic functions. Recent studies have uncovered a DNA-binding function of SOD1 [120–122]. The new function calls for a reexamination of this conserved protein, which may provide evidence that some variants are causative for ALS due to their loss of function.

Whether ALS-associated *SOD1* mutations predominantly lead to a loss of function or a gain of toxic function continues to be a central question in ALS research [123, 124]. In the following, we will review the molecular alterations of SOD1 caused by ALS-related variants.

#### Dismutase activity of variants

Bowling et al. initially proposed that decreased SOD1 activity might lead to elevated free radical levels, resulting in increased oxidative damage [125]. Their study reported a 38.8% reduction in cytosolic SOD (mainly SOD1) activity in post-mortem brain tissues of ALS patients with *SOD1* mutations (mutation in exon1; specific variant not provided) compared to controls. Although a supplement including recombinant SOD1 was suggested based on the loss-of-function hypothesis, no benefits were observed in two fALS patients [126]. There was no correlation between enzyme activity and the onset time or clinical forms of the disease [127].

To examine this hypothesis more rigorously, we compiled data from various studies on SOD1 activity in human tissues (Additional file 1: Table S4). Erythrocytes were predominantly studied for SOD1 activity due to the sample accessibility, with a noteworthy exception for the impact of SOD2 on mitochondria. The patients with different variants showed varying degrees of SOD1 activity decrease. Importantly, this reduction did not show a clear correlation with the severity of ALS.

Heterozygous carriers of p.C112Wfs*11, who have a markedly reduced enzyme activity compared to wild-type controls, show no overt neurologic phenotype [128, 129], further challenging the hypothesis.

#### SOD1 protein levels

SOD1 is ubiquitously expressed in human cells and its expression is regulated by basal transcription factors [113]. SOD1 immunoreactivity has been observed in the neural system, including motor neurons, small neurons, some astrocytes in the white matter, and ependymal cells in the central canal. The most intense staining was found in the cell bodies of large motor neurons in mice and humans [130].

ALS patients with SOD1 mutations have a noticeable decrease in SOD1 protein levels compared to normal controls (Additional file 1: Table S5). Remarkably, the mutant SOD1 species consistently have lower levels than wild-type SOD1 protein in heterozygotes. For instance, the total SOD1 protein level in erythrocytes from ALS patients carrying the heterozygous I114T mutation was 49% and 29% of that in controls, as measured by enzyme-linked immunosorbent assay [131] and liquid chromatography electrospray ionization mass spectrometry, respectively [132].

The reduced levels of SOD1 proteins, especially the mutant species in heterozygotes, suggest potential differences in the stability, turnover, or PTMs between mutant and wild-type SOD1 proteins.

Besides amino acid mutations, alterations within the regulatory regions of the *SOD1* gene have been implicated in the ALS pathogenesis. Notably, the c.-109A > G mutation within the TATA box has been found to decrease mRNA expression and protein levels of SOD1 [87]. Another notable case involves a 50-bp deletion located at 1684 bp upstream of the ATG starting codon in the *SOD1* promoter, leading to a reduction in *SOD1* gene expression [133]. A subsequent study on a Swedish cohort of 512 ALS patients and 354 controls revealed a statistically significant decreasing trend in SOD1 enzymatic activity associated with this allele. Interestingly, the frequencies of the heterozygous and homozygous genotypes were comparable in both the ALS and control cohorts [134]. Although this study identified a decreasing trend in enzymatic activity, a definitive association between this specific deletion and ALS pathogenesis was not conclusive. The association between mutations that affect SOD1 protein levels and the occurrence of ALS is still on debate.

#### Half-lives of mutant SOD1 proteins

SOD1 is a stable protein with a half-life of 183 h measured by metabolic pulse labelling in non-synchronized NIH3T3 mouse fibroblasts, in contrast to the median half-life of 46 h observed for all proteins. Additionally, *SOD1* mRNA exhibits a half-life of 15 h, longer than the median of 9 h observed for all mRNAs [135]. Noteworthy, in vivo kinetic approach revealed different turnover rates of SOD1 in different tissues in rats, with a slow turnover in the central nervous system [136].

ALS-related SOD1 variants exhibited decreased half-lives in transiently transfected monkey cells as examined with a radiolabeling technique (Additional file 1: Table S6). A consistent outcome was achieved for SOD1 A5V using the photoconvertible green fluorescent protein Dendra2 [137]. Notably, in cells with aggregates, the turnover rate was found to be slower compared to that in cells without aggregates. In a rat model carrying *SOD1* G94A, there was a significant acceleration in the turnover rate detected in CSF and spinal cord [136]. Further supporting these findings, stable isotope labeling in ALS patients revealed a consistent result of an accelerated half-life of A5V species in the CSF of affected individuals [138].

The fast degradation of the mutant species involves both autophagy and the ubiquitin–proteasome pathway, which serve to remove misfolded or oxidized proteins [139, 140]. RNF19A (also known as Dorfin), which is identified as a RING-finger type ubiquitin ligase and a juxtanuclearly located E3 ubiquityl ligase, plays a key role in the ubiquitylation of various SOD1 mutants from fALS patients, thereby facilitating degradation of mutant SOD1 [141, 142]. Intriguingly, these variants are proposed to subsequently inhibit the proteasome, leading to selective motor neuron death [143]. The failure of protein quality control has been proposed as a molecular phenotype in ALS [144]. However, the alteration of proteasomal function was not observed in neuronal cell lines in subsequent examination [145]. Another hypothesis is that the mutated SOD1 tends to increase proteasome activity to clear toxic proteins, but the increased proteasome activity may lead to proteostatic exhaustion on a broader scale, which contributes to the pathology of ALS [80, 146].

#### Aggregates in the neural system of *SOD1*-related ALS

Protein aggregates in the neural system are pathological characteristics of neurodegenerative diseases, such as beta-amyloid in Alzheimer's disease and alpha-synuclein in Parkinson's disease, as well as SOD1 [147], TARDBP and FUS [148–150] in ALS. Pathogenic variants show disruptions in protein folding, and misfolding of these species has been identified as key contributors to ALS pathogenesis. Currently, there is no cure for ALS, while strategies directly targeting SOD1, such as RNA interference (RNAi) against mutated SOD1 mRNA and antibodies targeting SOD1 aggregates, have been developed and show promising therapeutic potential against ALS.

A multitude of intracellular aggregates has been reported in ALS with different SOD1 variants (Additional file 1: Table S7), encompassing ubiquitinated inclusions prevalent in lower motor neurons of the spinal cord and brainstem. These inclusions exhibit diverse morphologies, ranging from ‘skein-like inclusions’, ‘Lewy body-like hyaline inclusions’ or a hybrid of both.

Another characteristic aggregate in ALS is the hyaline conglomerate inclusions, which are large multifocal accumulations composed of phosphorylated and non-phosphorylated neurofilament subunits (pNF and NF), along with other sequestered cytoplasmic proteins and organelles [151]. In vitro aggregation propensity has been evaluated with diverse SOD1 variants [152, 153]. The complex spectrum of intracellular aggregates highlights the diverse and complex pathological manifestations observed in ALS.

Remarkably, SOD1 aggregates have been found to bind amyloid-sensitive dyes such as Congo red or thioflavin T and S [80]. In contrast to trimeric SOD1, it has been observed that large SOD1 aggregates do not affect cell viability in a model of ALS [154, 155].

Key aspects in protein aggregation, including the spatiotemporal dynamics of early amyloid formation, the diversity of oligomers, the participation of on-pathway and off-pathway species, and the true origins of toxicity in the disease, remain subjects of debate [156].

## Current therapeutic strategies targeting SOD1

Current therapeutic strategies used to mitigate the impact of toxic misfolded proteins and aggregates in the context of ALS include: (1) lowering SOD1 protein levels to reduce the extent of its aggregation; (2) using the RNAi method to degrade mutant mRNA; (3) using antibodies to degrade mutant protein species; and (4) using genome editing techniques to correct the mutated nucleotide.

Pyrimethamine, an antimalarial drug, has been found to lower the SOD1 protein levels in the CSF of 2 ALS patients [157], which was confirmed later in a larger cohort of 32 patients [157, 158].

Antisense oligonucleotides (ASOs) have emerged as a promising strategy to downregulate SOD1 with enhanced stability, prolonged effect and reduced off-target effects. Richard et al. found that ASOs targeting *SOD1* decrease both protein levels and mRNA expression throughout the brain and spinal cord in rats [159]. ISIS 333611 which targets c.19–38 of wild-type *SOD1*, now renamed Tofersen (BIIB067), has entered clinical trials [160] and shown promising results [161–165]. On April 25, 2023, it was approved in the USA for the treatment of adult ALS patients carrying *SOD1* mutations [166]. In addition, 3 ASOs targeting 3’ UTR designed by Miller et al. have shown better therapeutic potential than the ISIS 333611 in mice carrying *SOD1* G94A .

Additionally, RfxCas13d, guided by the transactivating clustered regularly interspaced short palindromic repeats (CRISPR) RNA (crRNA) for mRNA degradation, has been specifically designed to silence *SOD1* and demonstrated high efficiency in *SOD1* knockdown [167]. Early studies (since 2003) with RNAi targeting mRNA of mutated *SOD1* using small interfering RNAs (siRNAs) and short hairpin RNAs were performed on models with A5V, G86R or G94A mutation [169–175]. To achieve higher permeability of the blood–brain barrier, adeno-associated virus (AAV) such as AAV6 and AAV9 have been used as viral vectors for packaging [176–180]. Chemically modified siRNAs are also showing promise [181].

A preclinical study has been conducted on anti-SOD1 nanobodies [180]. An epitope of Derlin-1, which selectively binds pathogenic SOD1, showed potentials to direct lysosomal degradation of the misfolded SOD1 protein in both cellular and mouse models [182]. Expression of an AAV-mediated antibody that blocks the β6/β7 loop epitope in the central nervous system of mice carrying human *SOD1* G38R, reduced the accumulation of misfolded SOD1.

Gene editing techniques, including genome editing [183] and base editing [184], have been successfully used in mouse models. The spectrum of therapeutic strategies mirrors the dynamic and evolving landscape of treatments for ALS. There is a need for further exploration of specific targeting of mutated species rather than a general reduction in SOD1 levels. This recommendation is particularly relevant given the finding that most of the SOD1 proteins in heterozygous patients consists of the wild-type species, as described in the aforementioned section.

The insights derived from these diverse strategies not only increase our understanding of ALS, but also lay the groundwork for potential combination therapies that may provide synergistic effects.

## Conclusions

In conclusion, our review sheds light on the complexities of ALS by focusing on the SOD1 protein. Acknowledging the limitations of our data derived from previous publications, not all identified SOD1 variants have clear pathogenicity. Notably, certain rare variants may not be linked to ALS, as SOD1 polymorphisms exist in the broader population without disease association [80, 185].

Besides SOD1, other proteins, including RNA-binding proteins such as TARDBP and FUS, as well as cytoskeletal proteins such as PFN1, contribute significantly to ALS pathogenesis. In addition to the monogenic inheritance pattern, an oligogenic inheritance model of ALS has been proposed [186, 187], which may account for the observed clinical heterogeneity. Gentile et al. characterized a small cohort of ALS patients carrying the D91A variant and discovered that all 7 patients also harbored variants of other ALS-related genes [188]. Further studies are needed to unravel the multifaceted interplay of genetic and environmental factors, protein aggregation, and cellular dysfunction that together drive ALS onset and progression.

Our review has provided insights into the geographical distribution, the clinical phenotype variations, and the structural impact of *SOD1* variants. This not only serves as a foundation for further research, but also hints at potential therapeutic targets, particularly in cases where protein structure alteration is a prevalent factor. While current therapies predominantly target the overall SOD1 levels, tailored therapies targeting specific mutant variants represent an exciting avenue for ALS treatment.

### Supplementary Information

**Additional file 1:** **Table S1.** SOD1 variant frequencies around the world in large cohorts. **Table S2.** Age of onset for ALS patients carrying different SOD1 variants. **Table S3.** Disease duration for ALS patients carrying different SOD1 variants. **Table S4.** Activity of mutant SOD1 in human tissues. **Table S5.** Protein level of mutant SOD1 in human tissues. **Table S6.** Half-lives of mutant SOD1 proteins. **Table S7.** Histological analysis of aggregations.

## Data Availability

The datasets used and/or analysed during the current study are available from the corresponding authors on reasonable request.

## Abbreviations

ALS: Amyotrophic lateral sclerosis
fALS: Familial ALS
sALS: Sporadic ALS
SNP: Single nucleotide polymorphism
UTR: Untranslated region
PTMs: Post-translational modifications
CSF: Cerebrospinal fluid
NF: Neurofilament
RNAi: RNA interference
ASO: Antisense oligonucleotide
siRNA: Small interfering RNA
AAV: Adeno-associated virus
