# Supplementary material for: Variability in SOD1-associated amyotrophic lateral sclerosis: geographic patterns, clinical heterogeneity, molecular alterations, and therapeutic implications
Source: Transl Neurodegener. 2024 May 29;13:28. doi: 10.1186/s40035-024-00416-x (PMC11138100; doi:10.1186/s40035-024-00416-x)
Supplement: Supplementary file 1 — Additional file 1: Table S1. SOD1 variant frequencies around the world in large cohorts. Table S2. Age of onset for ALS patients carrying different SOD1 variants. Table S3. Disease duration for ALS patients carrying different SOD1 variants. Table S4. Activity of mutant SOD1 in human tissues. Table S5. Protein level of mutant SOD1 in human tissues. Table S6. Half-lives of mutant SOD1 proteins. Table S7. Histological analysis of aggregations. [file 40035_2024_416_MOESM1_ESM.docx]

Table S1. *SOD1* variant frequencies around the world in large cohorts

| Country | Centers | Year | Cohort | Time period | Freq. in fALS | Freq. in sALS | Method | Ref. |
| --- | --- | --- | --- | --- | --- | --- | --- | --- |
| USA | Neuromuscular Clinic, Massachusetts General Hospital | 1997 | 395 | 1985 - Dec 1995 | 49.1% (194/395) | - | - | (1) |
| Italy | five Italian Departments of Neurology (Siena, Genova, Milano, Pisa and Cagliari) | 2005 | 264 | 1998 - 2002 | 17.9% (7/39) | 0 (0/225) | Sanger sequencing for all exons | (2) |
| Germany | Departments of Medical Genetics of the University of Giessen and Ulm | 2010 | 217 | 1998 - 2008 | 13% (28/217) | - | All exons and 30–50 bp flanking intronic sequences | (3) |
| Russia | Research Centre of Neurology, Moscow | 2015 | 208 | 2008 - 2013 | 44% (4/9) | 3.5% (7/199) | All exons and at least 50 bp flanking intronic sequences | (4) |
| USA | - | 2015 | 2874 | - | 12% | 1.5% | Exon sequencing | (5) |
| China | Department of Neurology, Xiangya Hospital | 2016 | 173 | - | 20% (3/15) | 1.9% (3/158) | Sanger sequencing for all exons | (6) |
| Japan | Japanese Consortium for Amyotrophic Lateral Sclerosis | 2016 | 508 | - | 35.9% (14/39) | 2.3% (11/469) | Sanger sequencing; Custom panel for all exons | (7) |
| China | Department of Neurology at the West China Hospital of Sichuan University | 2017 | 499 | - | 25% (3/12) | 1.03% (5/487) | Sanger sequencing for all exons and introns | (8) |
| Germany | German Clinical ALS Research Centres in Ulm, Berlin, Bochum,Essen, Hannover, Jena, Würzburg, Aachen and Munich | 2018 | 382 | 1995 - 2016 | 12.3% (47/382) | - | WES | (9) |
| China | Second Affiliated Hospital, Zhejiang University School of Medicine, First Affiliated Hospital, Fujian Medical University, and Huashan Hospital, Fudan University | 2019 | 45 | - | 50% (12/24) | 0 (0/21) | Custom panel for all exons and flanking regions | (10) |
| China | Department of Neurology, Peking University Third Hospital | 2019 | 1082 | 2007 – 2013 | 29.6% (47/159) | 2.1% (19/923) | Sanger sequencing for all exons and introns | (11) |
| USA | CReATe, FALS and ALSGENS consortia | 2019 | 3864 | - | - | 0.5% (21/3864) | WES | (12) |
| China | Department of Neurology of the First Affiliated Hospital of Sun Yat-Sen University | 2020 | 268 | Dec 2015 - Jul 2019 | 6.7% (1/15) | 1.6% (4/253) | Custom panel for all exons and the flanking regions (~ 50 bp) of the splice junctions surrounding the exons | (13) |
| Italy | Piemonte and Valle d’Aosta Register for ALS(14) | 2020 | 1410 | 2005 - 2015 | - | 2.6% (37/1410) | All the coding exons and 50 bp of the flanking intron–exon  boundaries | (15) |
| Brazil | 6 Brazilian neuromuscular reference centers | 2021 | 107 | 2015 - 2019 | 7.5% (8/107) | - | WES | (16) |
| China | Second Affiliated Hospital of Zhejiang University School of Medicine | 2021 | 129 | May 2017 - Apr 2021 | 66.7% (10/15) | 1.8% (2/114) | WES | (17) |
| Italy | Clinica Neurologica, Bellaria Hospital | 2021 | 330 | 2010 - 2019 | 9.5% (8/84) | 3.3% (8/246) | Sequencing for all exons | (18) |
| China | Department of Neurology, Xiangya Hospital | 2021 | 753 | Apr 2013 - Nov 2020 | 13% (8/62) | 2.75% (19/691) | WES | (19) |
| South Africa | ALS clinic at Groote Schuur Hospital in Cape Town | 2022 | 103 | 2014 - 2019 | 33% (1/3) | 0.4% (4/100) | WES | (20) |
| Norway | 17 neurological departments in Norway | 2022 | 279 | Aug 2019 - Aug 2021 | 28.1% (9/32) | 0.4% (1/247) | WES (Human Core Exome EF Multiplex kit) | (21) |
| Poland | Department of Neurology, the Medical University of Warsaw | 2022 | 915 | 2006 - 2018 | 21.1% (12/57) | 2.3% (20/855) | All exons with flanking intronic regions | (22) |
| China | Department of Neurology of West China Hospital | 2022 | 1587 | Dec 2010 - Jun 2019 | 21.9% (14/64) | 1.9% (29/1523) | WES | (23) |
| Spain | ALS Genetic Spanish Consortium (ALSGESCO) | 2022 | 5252 | - | 17.7% (116/656) | 0.5% (23/4596) | - | (24) |

Table S2. Age of onset for ALS patients carrying different *SOD1* variants

| Variant | Total reported cases | Mean age of onset (year) | Reported cases with detailed patient information | variance | Coefficient of dispersion | skewness |
| --- | --- | --- | --- | --- | --- | --- |
| Total | 1436 | 48.53±12.92 | 1087 | 166.82 | 0.27 | 0.06 |
| A5V | 184 | 48.72±11.85 | 53 | 140.49 | 0.24 | -0.56 |
| D91A_hom | 100 | 45.57±12.87 | 100 | 165.76 | 0.28 | 0.50 |
| D91A | 69 | 51.90±11.24 | 65 | 126.25 | 0.22 | 0.33 |
| H47R | 54 | 47.59±8.78 | 42 | 77.06 | 0.19 | 0.00 |
| L145F | 49 | 52.08±13.61 | 40 | 185.30 | 0.26 | -0.32 |
| I114T | 46 | 57.70±10.59 | 34 | 112.07 | 0.18 | -0.53 |
| R116G | 43 | 58.88±10.91 | 24 | 119.00 | 0.19 | -0.42 |
| E101G | 41 | 46.28±6.88 | 9 | 47.30 | 0.16 | 0.36 |
| L85F | 38 | 41.68±9.72 | 36 | 94.56 | 0.23 | -0.02 |
| E101K | 28 | 35.00±4.31 | 12 | 18.56 | 0.12 | -0.26 |
| K4E | 28 | 54.28±7.74 | 13 | 59.98 | 0.14 | 0.83 |
| G42S | 27 | 46.89±11.39 | 21 | 129.83 | 0.24 | -0.82 |
| I113T | 24 | 55.17±10.99 | 23 | 120.84 | 0.20 | 0.15 |
| L145S | 23 | 43.31±12.31 | 10 | 151.66 | 0.31 | 0.26 |
| C112Y | 22 | 47.79±13.73 | 21 | 188.44 | 0.28 | -0.08 |
| G94A | 22 | 46.85±9.05 | 8 | 81.86 | 0.20 | 0.74 |
| V149G | 21 | 44.98±8.08 | 12 | 65.27 | 0.19 | -0.82 |
| H44R | 18 | 48.63±8.07 | 10 | 65.08 | 0.16 | 1.29 |
| N87S | 17 | 55.18±13.92 | 16 | 193.65 | 0.25 | 0.00 |
| L127S | 14 | 52.93±15.23 | 14 | 232.07 | 0.29 | -0.02 |
| G94D | 14 | 44.28±16.01 | 12 | 256.30 | 0.37 | -0.17 |
| G86R | 14 | 53.32±11.69 | 4 | 136.55 | 0.24 | 0.15 |
| G38R | 13 | 33.61±8.43 | 11 | 71.04 | 0.25 | -0.15 |
| A5T | 13 | 45.70±13.41 | 7 | 179.83 | 0.33 | -0.42 |
| c.358-10T>G | 13 | 61.62±12.16 | 13 | 147.78 | 0.20 | 0.80 |
| G42D | 13 | 47.08±7.47 | 11 | 55.80 | 0.16 | 0.18 |
| C7S | 12 | 47.83±9.93 | 12 | 98.64 | 0.21 | 0.67 |
| V48A | 11 | 52.50±7.15 | 10 | 51.19 | 0.14 | 0.12 |
| H49R | 10 | 46.10±11.14 | 10 | 124.09 | 0.24 | -0.29 |
| G94V | 9 | 42.11±14.45 | 7 | 208.86 | 0.35 | -0.58 |
| G94S | 9 | 50.56±11.49 | 9 | 132.02 | 0.23 | -0.27 |
| L39V | 9 | 40.63±8.58 | 7 | 73.66 | 0.20 | 0.46 |
| G148D | 9 | 44.08±12.07 | 9 | 145.72 | 0.27 | 0.53 |
| G128Gfs*7 | 9 | 53.44±16.51 | 9 | 272.69 | 0.31 | -0.60 |
| G17A | 8 | 34.88±8.18 | 7 | 66.98 | 0.23 | 0.75 |
| P67S | 8 | 46.80±13.96 | 8 | 194.84 | 0.30 | 0.03 |
| S135N | 8 | 54.10±13.74 | 8 | 188.92 | 0.25 | 0.93 |
| V15M | 8 | 45.50±20.39 | 7 | 415.85 | 0.46 | -0.21 |
| L107V | 7 | 39.14±7.13 | 6 | 50.81 | 0.17 | -0.22 |
| F21C | 7 | 50.71±11.04 | 7 | 121.92 | 0.22 | 1.80 |
| L127* | 7 | 46.30±11.94 | 5 | 142.61 | 0.27 | -1.23 |
| G142A | 7 | 44.27±13.33 | 5 | 177.70 | 0.31 | -0.81 |
| I113M | 7 | 46.49±11.54 | 7 | 133.22 | 0.25 | -0.47 |
| S106L | 7 | 43.91±9.04 | 7 | 81.64 | 0.21 | -1.26 |
| A90V | 7 | 50.57±17.89 | 7 | 319.96 | 0.35 | -0.92 |
| D110Y | 6 | 57.67±3.64 | 6 | 13.22 | 0.06 | -0.50 |
| D102G | 6 | 42.67±4.38 | 6 | 19.22 | 0.10 | 0.03 |
| I150T | 6 | 43.67±5.15 | 5 | 26.56 | 0.12 | 0.59 |
| L107F | 6 | 44.47±5.99 | 4 | 35.93 | 0.13 | 0.13 |
| A5S | 6 | 32.33±7.59 | 6 | 57.56 | 0.23 | -0.07 |
| G73S | 6 | 48.27±15.49 | 6 | 239.96 | 0.32 | 0.39 |
| I105F | 6 | 52.00±11.38 | 4 | 129.50 | 0.24 | -0.17 |
| D77V | 5 | 45.92±1.97 | 5 | 3.87 | 0.04 | 0.10 |
| G17C | 5 | 51.40±3.01 | 5 | 9.04 | 0.06 | 0.54 |
| H121Q | 5 | 50.76±8.06 | 5 | 65.03 | 0.16 | 0.09 |
| N66S | 5 | 47.80±11.14 | 5 | 124.16 | 0.23 | 1.16 |
| A96T | 5 | 44.80±12.02 | 5 | 144.56 | 0.27 | 0.05 |
| L118V | 5 | 37.80±2.93 | 5 | 8.56 | 0.08 | 0.87 |
| C7F | 5 | 51.20±7.00 | 5 | 48.96 | 0.14 | -0.11 |
| D102N | 4 | 37.25±11.12 | 4 | 123.69 | 0.30 | 0.69 |
| D77Y | 4 | 45.00±4.06 | 4 | 16.50 | 0.09 | -0.09 |
| D12Y | 4 | 52.75±8.26 | 4 | 68.19 | 0.16 | -0.44 |
| c.240-7T>G | 4 | 42.00±0.00 | 4 | 0.00 | 0.00 | 0.00 |
| c.239+34A>C | 4 | 46.50±16.37 | 4 | 267.88 | 0.35 | 0.27 |
| N66T | 4 | 70.00±7.52 | 4 | 56.50 | 0.11 | -1.14 |
| V32A | 4 | 48.60±1.33 | 4 | 1.78 | 0.03 | -0.82 |
| G13R | 4 | 52.75±13.53 | 4 | 183.19 | 0.26 | -0.97 |
| C7W | 4 | 67.00±8.40 | 4 | 70.50 | 0.13 | -0.92 |
| I114F | 4 | 53.00±4.74 | 4 | 22.50 | 0.09 | 0.98 |
| T138A | 4 | 59.50±8.20 | 4 | 67.25 | 0.14 | 0.36 |
| G115A | 4 | 35.50±3.77 | 4 | 14.25 | 0.11 | -1.12 |
| G94C | 3 | 44.67±8.96 | 3 | 80.22 | 0.20 | -0.71 |
| G73D | 3 | 46.20±2.97 | 3 | 8.82 | 0.06 | 0.71 |
| E134* | 3 | 43.67±4.19 | 3 | 17.56 | 0.10 | -0.45 |
| D91A&D97N | 3 | 38.67±6.18 | 3 | 38.22 | 0.16 | -0.65 |
| G142* | 3 | 39.33±4.11 | 3 | 16.89 | 0.10 | -0.24 |
| S135T | 3 | 53.67±6.24 | 3 | 38.89 | 0.12 | 0.38 |
| A90T | 3 | 52.13±7.03 | 3 | 49.44 | 0.13 | 0.48 |
| A146T | 3 | 56.00±4.24 | 3 | 18.00 | 0.08 | -0.71 |
| V120F | 3 | 39.40±9.63 | 3 | 92.65 | 0.24 | 0.70 |
| V119L | 3 | 35.67±3.30 | 3 | 10.89 | 0.09 | -0.71 |
| G73C | 3 | 70.00±0.82 | 3 | 0.67 | 0.01 | 0.00 |
| D125G | 3 | 60.67±6.60 | 3 | 43.56 | 0.11 | -0.29 |
| E133del | 3 | 48.00±5.35 | 3 | 28.67 | 0.11 | 0.27 |
| L9Q | 3 | 43.67±8.99 | 3 | 80.89 | 0.21 | 0.11 |
| N140H | 3 | 59.10±0.70 | 3 | 0.49 | 0.01 | 0.21 |
| N140K | 3 | 51.67±11.61 | 3 | 134.89 | 0.22 | 0.67 |
| G11V | 3 | 29.67±4.64 | 3 | 21.56 | 0.16 | 0.49 |
| Q23L | 3 | 47.00±1.50 | 2 | 2.25 | 0.03 | 0.00 |
| V6L | 3 | 56.43±14.71 | 3 | 216.24 | 0.26 | -0.60 |
| E22G | 3 | 47.33±7.54 | 3 | 56.89 | 0.16 | 0.71 |
| G142E | 3 | 45.67±3.77 | 3 | 14.22 | 0.08 | 0.71 |
| I152T | 3 | 48.00±0.00 | 2 | 0.00 | 0.00 | 0.00 |
| C147R | 2 | 48.50±9.50 | 2 | 90.25 | 0.20 | 0.00 |
| L127Gfs*6 | 2 | 41.50±0.50 | 2 | 0.25 | 0.01 | 0.00 |
| K92Rfs*9 | 2 | 67.00±0.00 | 2 | 0.00 | 0.00 | 0.00 |
| D84G | 2 | 40.50±8.50 | 2 | 72.25 | 0.21 | 0.00 |
| L145S_hom | 2 | 24.50±6.50 | 2 | 42.25 | 0.27 | 0.00 |
| L39R | 2 | 43.20±10.80 | 2 | 116.64 | 0.25 | 0.00 |
| L85V | 2 | 34.00±5.00 | 2 | 25.00 | 0.15 | 0.00 |
| N140D | 2 | 57.00±0.00 | 1 | 0.00 | 0.00 | 0.00 |
| N20S | 2 | 46.50±5.50 | 2 | 30.25 | 0.12 | 0.00 |
| N87D | 2 | 55.00±0.00 | 1 | 0.00 | 0.00 | 0.00 |
| N87K | 2 | 58.00±5.00 | 2 | 25.00 | 0.09 | 0.00 |
| P67A | 2 | 52.85±11.75 | 2 | 138.06 | 0.22 | 0.00 |
| P75S | 2 | 58.50±0.50 | 2 | 0.25 | 0.01 | 0.00 |
| Q23R | 2 | 37.50±0.50 | 2 | 0.25 | 0.01 | 0.00 |
| T138R | 2 | 48.50±0.50 | 2 | 0.25 | 0.01 | 0.00 |
| V119M | 2 | 77.50±4.50 | 2 | 20.25 | 0.06 | 0.00 |
| c.-109A>G | 2 | 56.00±14.00 | 2 | 196.00 | 0.25 | 0.00 |
| c.169+50delAACAGTA | 2 | 64.00±1.00 | 2 | 1.00 | 0.02 | 0.00 |
| K129Kfs*4 | 2 | 41.50±0.50 | 2 | 0.25 | 0.01 | 0.00 |
| A141A | 2 | 62.50±11.50 | 2 | 132.25 | 0.18 | 0.00 |
| D84H | 2 | 57.60±0.30 | 2 | 0.09 | 0.01 | 0.00 |
| H72Y | 2 | 38.00±19.00 | 2 | 361.00 | 0.50 | 0.00 |
| F46C | 2 | 63.00±4.00 | 2 | 16.00 | 0.06 | 0.00 |
| E50K | 2 | 46.50±0.50 | 2 | 0.25 | 0.01 | 0.00 |
| E41G | 2 | 50.00±14.00 | 2 | 196.00 | 0.28 | 0.00 |
| G148N | 2 | 56.50±1.50 | 2 | 2.25 | 0.03 | 0.00 |
| G17S | 2 | 24.00±0.00 | 2 | 0.00 | 0.00 | 0.00 |
| E122G | 2 | 70.00±0.00 | 2 | 0.00 | 0.00 | 0.00 |
| D102H | 2 | 52.00±0.00 | 2 | 0.00 | 0.00 | 0.00 |
| G94R | 2 | 35.00±1.00 | 2 | 1.00 | 0.03 | 0.00 |
| D126N | 2 | 53.50±11.50 | 2 | 132.25 | 0.21 | 0.00 |
| D125Tfs*24 | 2 | 55.00±0.00 | 2 | 0.00 | 0.00 | 0.00 |
| C7G | 2 | 49.50±3.50 | 2 | 12.25 | 0.07 | 0.00 |
| I150V | 2 | 42.00±0.00 | 1 | 0.00 | 0.00 | 0.00 |
| H81Y | 2 | 46.50±4.50 | 2 | 20.25 | 0.10 | 0.00 |
| F21V | 1 | 38.00±0.00 | 1 | 0.00 | 0.00 | 0.00 |
| E134K | 1 | 62.00±0.00 | 1 | 0.00 | 0.00 | 0.00 |
| D91N | 1 | 71.00±0.00 | 1 | 0.00 | 0.00 | 0.00 |
| E134V | 1 | 46.00±0.00 | 1 | 0.00 | 0.00 | 0.00 |
| c.240-3T>C | 1 | 25.00±0.00 | 1 | 0.00 | 0.00 | 0.00 |
| D84N | 1 | 67.00±0.00 | 1 | 0.00 | 0.00 | 0.00 |
| S60S | 1 | 77.00±0.00 | 1 | 0.00 | 0.00 | 0.00 |
| S69P_hom | 1 | 25.00±0.00 | 1 | 0.00 | 0.00 | 0.00 |
| F46S | 1 | 57.00±0.00 | 1 | 0.00 | 0.00 | 0.00 |
| S108Lfs*15 | 1 | 64.00±0.00 | 1 | 0.00 | 0.00 | 0.00 |
| S106delSL | 1 | 52.00±0.00 | 1 | 0.00 | 0.00 | 0.00 |
| G11A | 1 | 46.00±0.00 | 1 | 0.00 | 0.00 | 0.00 |
| R80S | 1 | 38.00±0.00 | 1 | 0.00 | 0.00 | 0.00 |
| A5F | 1 | 48.00±0.00 | 1 | 0.00 | 0.00 | 0.00 |
| c.-46C>T | 1 | 42.00±0.00 | 1 | 0.00 | 0.00 | 0.00 |
| V15G | 1 | 41.00±0.00 | 1 | 0.00 | 0.00 | 0.00 |
| E134G | 1 | 34.00±0.00 | 1 | 0.00 | 0.00 | 0.00 |
| E133* | 1 | 40.00±0.00 | 1 | 0.00 | 0.00 | 0.00 |
| A153P | 1 | 55.00±0.00 | 1 | 0.00 | 0.00 | 0.00 |
| V48F | 1 | 36.00±0.00 | 1 | 0.00 | 0.00 | 0.00 |
| G11R | 1 | 68.00±0.00 | 1 | 0.00 | 0.00 | 0.00 |
| c.170-7T>C | 1 | 56.00±0.00 | 1 | 0.00 | 0.00 | 0.00 |
| V6M | 1 | 71.00±0.00 | 1 | 0.00 | 0.00 | 0.00 |
| V88A | 1 | 51.00±0.00 | 1 | 0.00 | 0.00 | 0.00 |
| V95G | 1 | 39.00±0.00 | 1 | 0.00 | 0.00 | 0.00 |
| c.170-20T>C | 1 | 72.00±0.00 | 1 | 0.00 | 0.00 | 0.00 |
| D93G_hom | 1 | 54.00±0.00 | 1 | 0.00 | 0.00 | 0.00 |
| W33* | 1 | 41.00±0.00 | 1 | 0.00 | 0.00 | 0.00 |
| W33G | 1 | 40.00±0.00 | 1 | 0.00 | 0.00 | 0.00 |
| c.*249T>C | 1 | 46.00±0.00 | 1 | 0.00 | 0.00 | 0.00 |
| R70G | 1 | 57.10±0.00 | 1 | 0.00 | 0.00 | 0.00 |
| P67S&D91A | 1 | 66.00±0.00 | 1 | 0.00 | 0.00 | 0.00 |
| G128R | 1 | 60.00±0.00 | 1 | 0.00 | 0.00 | 0.00 |
| G128V | 1 | 32.70±0.00 | 1 | 0.00 | 0.00 | 0.00 |
| G38V | 1 | 45.00±0.00 | 1 | 0.00 | 0.00 | 0.00 |
| L85F_hom | 1 | 13.00±0.00 | 1 | 0.00 | 0.00 | 0.00 |
| G62R | 1 | 56.00±0.00 | 1 | 0.00 | 0.00 | 0.00 |
| L68P | 1 | 36.00±0.00 | 1 | 0.00 | 0.00 | 0.00 |
| H121R | 1 | 26.00±0.00 | 1 | 0.00 | 0.00 | 0.00 |
| D102E | 1 | 55.60±0.00 | 1 | 0.00 | 0.00 | 0.00 |
| L145Ffs*3 | 1 | 39.00±0.00 | 1 | 0.00 | 0.00 | 0.00 |
| H49E | 1 | 53.00±0.00 | 1 | 0.00 | 0.00 | 0.00 |
| H81R | 1 | 24.00±0.00 | 1 | 0.00 | 0.00 | 0.00 |
| L118V_hom | 1 | 44.00±0.00 | 1 | 0.00 | 0.00 | 0.00 |
| I100V | 1 | 59.00±0.00 | 1 | 0.00 | 0.00 | 0.00 |
| I113F | 1 | 68.00±0.00 | 1 | 0.00 | 0.00 | 0.00 |
| I150M | 1 | 55.00±0.00 | 1 | 0.00 | 0.00 | 0.00 |
| C58Y | 1 | 73.10±0.00 | 1 | 0.00 | 0.00 | 0.00 |
| K137* | 1 | 45.00±0.00 | 1 | 0.00 | 0.00 | 0.00 |
| L9V | 1 | 43.00±0.00 | 1 | 0.00 | 0.00 | 0.00 |
| N132Qfs*5 | 1 | 32.00±0.00 | 1 | 0.00 | 0.00 | 0.00 |
| N140* | 1 | 45.00±0.00 | 1 | 0.00 | 0.00 | 0.00 |
| G139E | 1 | 41.00±0.00 | 1 | 0.00 | 0.00 | 0.00 |
| G131Kfs*2 | 1 | 21.40±0.00 | 1 | 0.00 | 0.00 | 0.00 |
| P67T | 1 | 37.60±0.00 | 1 | 0.00 | 0.00 | 0.00 |
| I19del | 1 | 80.00±0.00 | 1 | 0.00 | 0.00 | 0.00 |
| G131delGNEE | 1 | 36.00±0.00 | 1 | 0.00 | 0.00 | 0.00 |
| P67R | 1 | 34.00±0.00 | 1 | 0.00 | 0.00 | 0.00 |
| P67L | 1 | 35.00±0.00 | 1 | 0.00 | 0.00 | 0.00 |
| A90F | 1 | 45.00±0.00 | 1 | 0.00 | 0.00 | 0.00 |
| G38C | 1 | 44.00±0.00 | 1 | 0.00 | 0.00 | 0.00 |
| G139V | 1 | 34.00±0.00 | 1 | 0.00 | 0.00 | 0.00 |
| N87I | 1 | 55.20±0.00 | 1 | 0.00 | 0.00 | 0.00 |
| G148C | 1 | 61.00±0.00 | 1 | 0.00 | 0.00 | 0.00 |
| G148S | 1 | 56.00±0.00 | 1 | 0.00 | 0.00 | 0.00 |
| G17H | 1 | 23.00±0.00 | 1 | 0.00 | 0.00 | 0.00 |
| G28delGP_hom | 1 | 51.00±0.00 | 1 | 0.00 | 0.00 | 0.00 |
| I152S | 1 | 50.30±0.00 | 1 | 0.00 | 0.00 | 0.00 |

Table S3. Disease duration for ALS patients carrying different *SOD1* variants

| Variant | Total reported cases | Mean disease duration (month) | Reported cases with detailed patient information | variance | Coefficient of dispersion | skewness |
| --- | --- | --- | --- | --- | --- | --- |
| total | 636 | 56.17±76.15 | 490 | 5799.42 | 1.23 | 2.70 |
| A5V | 110 | 12.90±7.80 | 36 | 60.77 | 0.53 | 0.96 |
| D91A_hom | 34 | 136.86±97.38 | 34 | 9482.72 | 0.71 | 0.74 |
| I114T | 29 | 45.10±35.28 | 21 | 1244.59 | 0.82 | 1.18 |
| E101G | 25 | 57.66±21.09 | 3 | 444.81 | 0.66 | 0.63 |
| L145F | 21 | 130.81±155.84 | 19 | 24287.06 | 1.19 | 1.78 |
| D91A | 19 | 125.71±80.26 | 19 | 6441.54 | 0.64 | 0.34 |
| G42S | 17 | 12.71±4.27 | 17 | 18.21 | 0.34 | -0.13 |
| L85F | 16 | 55.12±30.59 | 16 | 935.98 | 0.55 | 1.28 |
| H44R | 13 | 15.40±9.58 | 7 | 91.72 | 0.68 | 1.56 |
| H47R | 12 | 201.00±70.96 | 8 | 5034.75 | 0.36 | -0.68 |
| G86R | 12 | 67.50±27.00 | 2 | 729.00 | 0.60 | 0.00 |
| c.358-10T>G | 11 | 34.00±20.92 | 11 | 437.82 | 0.62 | 0.77 |
| C112Y | 10 | 46.77±21.14 | 10 | 447.02 | 0.45 | 0.74 |
| A5T | 10 | 20.21±14.23 | 4 | 202.43 | 0.60 | 0.85 |
| K4E | 8 | 81.00±52.90 | 8 | 2798.25 | 0.65 | 0.53 |
| I113T | 8 | 22.62±14.63 | 8 | 213.92 | 0.65 | 1.59 |
| G94A | 8 | 37.50±20.68 | 3 | 427.56 | 0.40 | 0.33 |
| N87S | 8 | 61.12±31.53 | 8 | 994.36 | 0.52 | 0.37 |
| C7S | 8 | 86.00±50.00 | 8 | 2500.00 | 0.58 | 0.61 |
| V149G | 7 | 24.00±0.00 | 1 | 0.00 | 0.00 | 0.00 |
| L107V | 7 | 24.40±23.20 | 6 | 538.42 | 0.89 | 1.50 |
| R116G | 6 | 19.45±9.51 | 6 | 90.35 | 0.49 | 0.52 |
| L127S | 6 | 81.33±34.98 | 6 | 1223.89 | 0.43 | -0.22 |
| G94S | 6 | 93.00±41.90 | 6 | 1756.00 | 0.45 | 0.12 |
| G148D | 6 | 13.80±5.15 | 6 | 26.53 | 0.37 | 0.96 |
| G128Gfs*7 | 6 | 19.67±9.41 | 6 | 88.56 | 0.48 | -0.40 |
| S135N | 6 | 39.17±43.33 | 6 | 1877.81 | 1.11 | 1.35 |
| G38R | 5 | 152.20±76.25 | 3 | 5813.56 | 0.70 | 0.62 |
| L39V | 5 | 21.58±6.87 | 5 | 47.15 | 0.32 | 0.05 |
| D102G | 5 | 32.20±26.88 | 5 | 722.56 | 0.83 | 1.22 |
| G17C | 5 | 56.40±27.17 | 5 | 738.24 | 0.48 | 1.18 |
| H121Q | 5 | 38.84±10.92 | 5 | 119.14 | 0.28 | -0.01 |
| E101K | 4 | 299.00±123.45 | 4 | 15239.58 | 0.41 | -0.38 |
| P67S | 4 | 14.05±5.66 | 4 | 32.01 | 0.40 | -0.03 |
| I113M | 4 | 19.15±8.82 | 4 | 77.77 | 0.46 | 0.79 |
| S106L | 4 | 82.75±28.70 | 4 | 823.69 | 0.35 | 1.09 |
| A5S | 4 | 70.00±54.66 | 4 | 2988.00 | 0.78 | 0.62 |
| D77V | 4 | 225.00±81.99 | 4 | 6723.00 | 0.36 | -0.53 |
| c.240-7T>G | 4 | 19.75±0.43 | 4 | 0.19 | 0.02 | -1.15 |
| G115A | 4 | 28.75±8.98 | 4 | 80.69 | 0.31 | -0.84 |
| G42D | 3 | 29.40±17.42 | 3 | 303.39 | 0.59 | 0.70 |
| G94V | 3 | 104.00±34.41 | 3 | 1184.00 | 0.33 | -0.17 |
| L127* | 3 | 64.67±48.78 | 3 | 2379.56 | 0.75 | 0.56 |
| G73S | 3 | 13.17±2.25 | 3 | 5.06 | 0.17 | -0.68 |
| C7F | 3 | 12.33±0.47 | 3 | 0.22 | 0.04 | 0.71 |
| N66T | 3 | 25.00±9.20 | 3 | 84.67 | 0.37 | 0.70 |
| G142* | 3 | 29.00±22.55 | 3 | 508.67 | 0.78 | 0.54 |
| V119L | 3 | 65.67±33.25 | 3 | 1105.56 | 0.51 | -0.66 |
| G11V | 3 | 12.00±3.56 | 3 | 12.67 | 0.30 | -0.67 |
| V48A | 2 | 11.05±5.95 | 2 | 35.40 | 0.54 | 0.00 |
| H49R | 2 | 218.40±146.40 | 2 | 21432.96 | 0.67 | 0.00 |
| G17A | 2 | 78.00±30.00 | 2 | 900.00 | 0.38 | 0.00 |
| V15M | 2 | 60.50±48.50 | 2 | 2352.25 | 0.80 | 0.00 |
| I150T | 2 | 57.00±45.00 | 2 | 2025.00 | 0.79 | 0.00 |
| L107F | 2 | 18.20±9.20 | 2 | 84.64 | 0.51 | 0.00 |
| D102N | 2 | 36.00±0.00 | 2 | 0.00 | 0.00 | 0.00 |
| D77Y | 2 | 204.00±0.00 | 2 | 0.00 | 0.00 | 0.00 |
| C7W | 2 | 23.00±1.00 | 2 | 1.00 | 0.04 | 0.00 |
| I114F | 2 | 68.50±53.50 | 2 | 2862.25 | 0.78 | 0.00 |
| G73D | 2 | 63.45±0.45 | 2 | 0.20 | 0.01 | 0.00 |
| D91A&D97N | 2 | 222.00±114.00 | 2 | 12996.00 | 0.51 | 0.00 |
| G73C | 2 | 56.50±3.50 | 2 | 12.25 | 0.06 | 0.00 |
| D125G | 2 | 31.50±3.50 | 2 | 12.25 | 0.11 | 0.00 |
| E133del | 2 | 87.50±5.50 | 2 | 30.25 | 0.06 | 0.00 |
| N140H | 2 | 39.00±8.00 | 2 | 64.00 | 0.21 | 0.00 |
| C147R | 2 | 46.50±37.50 | 2 | 1406.25 | 0.81 | 0.00 |
| L127Gfs*6 | 2 | 20.50±3.50 | 2 | 12.25 | 0.17 | 0.00 |
| D84G | 2 | 53.50±16.50 | 2 | 272.25 | 0.31 | 0.00 |
| L145S_hom | 2 | 66.00±42.00 | 2 | 1764.00 | 0.64 | 0.00 |
| L39R | 2 | 17.15±7.15 | 2 | 51.12 | 0.42 | 0.00 |
| T138R | 2 | 10.50±3.50 | 2 | 12.25 | 0.33 | 0.00 |
| V119M | 2 | 57.00±39.00 | 2 | 1521.00 | 0.68 | 0.00 |
| c.169+50delAACAGTA | 2 | 51.00±27.00 | 2 | 729.00 | 0.53 | 0.00 |
| K129Kfs*4 | 2 | 69.00±27.00 | 2 | 729.00 | 0.39 | 0.00 |
| D84H | 2 | 17.40±4.10 | 2 | 16.81 | 0.24 | 0.00 |
| H72Y | 2 | 12.00±0.00 | 2 | 0.00 | 0.00 | 0.00 |
| G148N | 2 | 20.50±11.50 | 2 | 132.25 | 0.56 | 0.00 |
| D102H | 2 | 3.00±0.00 | 2 | 0.00 | 0.00 | 0.00 |
| G94R | 2 | 63.00±8.00 | 2 | 64.00 | 0.13 | 0.00 |
| C7G | 2 | 2.50±0.50 | 2 | 0.25 | 0.20 | 0.00 |
| G94D | 1 | 30.00±0.00 | 1 | 0.00 | 0.00 | 0.00 |
| G142A | 1 | 47.00±0.00 | 1 | 0.00 | 0.00 | 0.00 |
| A90V | 1 | 157.00±0.00 | 1 | 0.00 | 0.00 | 0.00 |
| D110Y | 1 | 72.00±0.00 | 1 | 0.00 | 0.00 | 0.00 |
| I105F | 1 | 250.80±0.00 | 1 | 0.00 | 0.00 | 0.00 |
| D12Y | 1 | 48.00±0.00 | 1 | 0.00 | 0.00 | 0.00 |
| V32A | 1 | 168.40±0.00 | 1 | 0.00 | 0.00 | 0.00 |
| G13R | 1 | 84.00±0.00 | 1 | 0.00 | 0.00 | 0.00 |
| G94C | 1 | 67.00±0.00 | 1 | 0.00 | 0.00 | 0.00 |
| E134* | 1 | 8.00±0.00 | 1 | 0.00 | 0.00 | 0.00 |
| S135T | 1 | 84.00±0.00 | 1 | 0.00 | 0.00 | 0.00 |
| A90T | 1 | 43.70±0.00 | 1 | 0.00 | 0.00 | 0.00 |
| A146T | 1 | 11.00±0.00 | 1 | 0.00 | 0.00 | 0.00 |
| V120F | 1 | 30.90±0.00 | 1 | 0.00 | 0.00 | 0.00 |
| L9Q | 1 | 8.00±0.00 | 1 | 0.00 | 0.00 | 0.00 |
| N140K | 1 | 10.00±0.00 | 1 | 0.00 | 0.00 | 0.00 |
| V6L | 1 | 46.00±0.00 | 1 | 0.00 | 0.00 | 0.00 |
| E22G | 1 | 73.00±0.00 | 1 | 0.00 | 0.00 | 0.00 |
| K92Rfs*9 | 1 | 12.00±0.00 | 1 | 0.00 | 0.00 | 0.00 |
| L85V | 1 | 66.00±0.00 | 1 | 0.00 | 0.00 | 0.00 |
| N87K | 1 | 26.00±0.00 | 1 | 0.00 | 0.00 | 0.00 |
| P75S | 1 | 65.00±0.00 | 1 | 0.00 | 0.00 | 0.00 |
| c.-109A>G | 1 | 60.00±0.00 | 1 | 0.00 | 0.00 | 0.00 |
| A141A | 1 | 113.00±0.00 | 1 | 0.00 | 0.00 | 0.00 |
| E41G | 1 | 36.00±0.00 | 1 | 0.00 | 0.00 | 0.00 |
| G17S | 1 | 70.00±0.00 | 1 | 0.00 | 0.00 | 0.00 |
| E122G | 1 | 168.00±0.00 | 1 | 0.00 | 0.00 | 0.00 |
| D126N | 1 | 24.00±0.00 | 1 | 0.00 | 0.00 | 0.00 |
| H81Y | 1 | 12.00±0.00 | 1 | 0.00 | 0.00 | 0.00 |
| F21V | 1 | 21.00±0.00 | 1 | 0.00 | 0.00 | 0.00 |
| D91N | 1 | 8.00±0.00 | 1 | 0.00 | 0.00 | 0.00 |
| E134V | 1 | 23.00±0.00 | 1 | 0.00 | 0.00 | 0.00 |
| S60S | 1 | 12.00±0.00 | 1 | 0.00 | 0.00 | 0.00 |
| S69P_hom | 1 | 36.00±0.00 | 1 | 0.00 | 0.00 | 0.00 |
| S106delSL | 1 | 31.00±0.00 | 1 | 0.00 | 0.00 | 0.00 |
| c.-46C>T | 1 | 66.00±0.00 | 1 | 0.00 | 0.00 | 0.00 |
| V15G | 1 | 20.00±0.00 | 1 | 0.00 | 0.00 | 0.00 |
| E134G | 1 | 54.00±0.00 | 1 | 0.00 | 0.00 | 0.00 |
| E133* | 1 | 15.00±0.00 | 1 | 0.00 | 0.00 | 0.00 |
| V6M | 1 | 40.00±0.00 | 1 | 0.00 | 0.00 | 0.00 |
| W33G | 1 | 70.00±0.00 | 1 | 0.00 | 0.00 | 0.00 |
| c.*249T>C | 1 | 30.00±0.00 | 1 | 0.00 | 0.00 | 0.00 |
| G128R | 1 | 7.00±0.00 | 1 | 0.00 | 0.00 | 0.00 |
| G128V | 1 | 10.40±0.00 | 1 | 0.00 | 0.00 | 0.00 |
| G38V | 1 | 13.00±0.00 | 1 | 0.00 | 0.00 | 0.00 |
| L85F_hom | 1 | 12.00±0.00 | 1 | 0.00 | 0.00 | 0.00 |
| H121R | 1 | 34.00±0.00 | 1 | 0.00 | 0.00 | 0.00 |
| D102E | 1 | 27.00±0.00 | 1 | 0.00 | 0.00 | 0.00 |
| L145Ffs*3 | 1 | 10.00±0.00 | 1 | 0.00 | 0.00 | 0.00 |
| H49E | 1 | 13.00±0.00 | 1 | 0.00 | 0.00 | 0.00 |
| H81R | 1 | 18.00±0.00 | 1 | 0.00 | 0.00 | 0.00 |
| C58Y | 1 | 30.50±0.00 | 1 | 0.00 | 0.00 | 0.00 |
| N132Qfs*5 | 1 | 31.00±0.00 | 1 | 0.00 | 0.00 | 0.00 |
| G139E | 1 | 18.00±0.00 | 1 | 0.00 | 0.00 | 0.00 |
| P67T | 1 | 58.80±0.00 | 1 | 0.00 | 0.00 | 0.00 |
| G139V | 1 | 20.00±0.00 | 1 | 0.00 | 0.00 | 0.00 |
| N87I | 1 | 41.60±0.00 | 1 | 0.00 | 0.00 | 0.00 |
| G148S | 1 | 8.00±0.00 | 1 | 0.00 | 0.00 | 0.00 |
| G17H | 1 | 36.00±0.00 | 1 | 0.00 | 0.00 | 0.00 |
| G28delGP_hom | 1 | 48.00±0.00 | 1 | 0.00 | 0.00 | 0.00 |
| I152S | 1 | 30.50±0.00 | 1 | 0.00 | 0.00 | 0.00 |

Table S4. Activity of mutant SOD1 in human tissues

| Variant | Activity | Compartments | Age of onset (year) | Disease duration (month) | Tissue | Method | Ref |
| --- | --- | --- | --- | --- | --- | --- | --- |
| A5V | 58% | 2 patients vs 31 controls | 51.5 | 14 | Red blood cell | Epinephrine autoxidation assay; SOD-525 kit | (25) |
| A5V | 59% | 2 patient vs 4 controls | 51.5 | 14 | Lymphoblastoid cell line | Xanthine oxidation assay | (25) |
| A5V | 57% | 3 patients vs 12 controls | 51.5 | 14 | Brain | Epinephrine autoxidation assay | (25) |
| A5V | 58% | 4 patients vs 25 controls | 50 | >14 | Erythrocyte | Epinephrine autoxidation assay | (26) |
| A5V | 34% | 4 patients vs 19 controls | 46 (age at death) | - | Brain area 4 | Epinephrine autoxidation assay | (27) |
| A5V | 57% | 4 patients vs 28 controls | 46 (age at death) | - | Brain area 40 | Epinephrine autoxidation assay | (27) |
| A5V | 44% | 4 patients vs 16 controls | 46 (age at death) | - | Cerebellum | Epinephrine autoxidation assay | (27) |
| A5V | 46% | 1 patient vs 4 controls | 62 | 16 | Erythrocyte | - | (28) |
| C7S | 74% | 5 patients vs 5 controls | 52 | 80 | Erythrocytes | Direct spectrophotometric method using KO_2_(29) | (30) |
| G28delGP | 70% | 1 patient vs 2 controls | 55 | 52 | Erythrocyte | Superoxide dismutase activity kit (900-157, Stressgen) | (31) |
| G28delGP_hom | 50% | 1 patient vs 2 controls | 51 | 48 | Erythrocyte | Superoxide dismutase activity kit (900-157, Stressgen) | (31) |
| G38R | 60% | 1 patient vs wild type | - | - | Lymphoblast cell lines | Nitroblue tetrazolium-based SOD staining method | (32) |
| L39V | 65% | 1 patient vs Families without mutation | 36.8 (age) | - | Red blood cell | SOD-525 kit | (33) |
| G42S | 69% | 9 patients vs 9 families without mutation | 43 (age) | - | Lymphoblasts | Xanthine oxidation assay | (34) |
| H47R | 47% | 1 patient vs 4 controls | 54 | >143 | Erythrocyte | - | (28) |
| S69P_hom | 17% | 1 patient vs 372 controls | 25 | 24 | Blood | Direct spectrophotometric method using KO_2_ | (35) |
| L85F | 91% | 4 patients vs 4 controls | 43 | 61 | Erythrocyte | Xanthine oxidation assay | (36) |
| L85F | 91% | 4 patients vs 11 controls | 43 | 61 | Lymphocyte | Xanthine oxidation assay | (36) |
| L85F | 81% | 3 patients vs 11 controls | 47 | 74 | Erythrocyte | Xanthine oxidation assay | (37) |
| G73S | 55% | 1 patient vs normal unaffected individuals | 47 | 48 | Erythrocyte | Direct spectrophotometric method using KO_2_ | (38) |
| N87S | 46% | 1 patient vs 4 controls | 77 | 24 | Erythrocyte | - | (28) |
| D91A_hom | 92% | 33 patients vs 66 controls | 55 (age) | - | Erythrocyte | Direct spectrophotometric method using KO_2_ | (39) |
| D91A_hom | 86% | 1 patient vs 4 controls | 52 | >324 | Erythrocyte | - | (28) |
| D91V | 41% | 1 patient vs 5 controls | 44 | - | Erythrocyte | Nitrite method | (40) |
| G94D | 63% | 6 patients vs 25 controls | 48 | >180 | Erythrocyte | Epinephrine autoxidation assay | (26) |
| G94A | 51% | 1 patients vs 4 controls | 42 | 47 | Erythrocyte | - | (28) |
| G94A | 74% | 1 patients vs 25 controls | 48 | >26 | Erythrocyte | Epinephrine autoxidation assay | (26) |
| G94S | 93% | 1 patient vs 5 controls | 33 | >180 | Fibroblast | Nitrite method | (41) |
| G94S | 99% | 1 patient vs 5 controls | 33 | >180 | Erythrocyte | Nitrite method | (41) |
| G94R | 32% | 1 family vs controls | - | - | Erythrocyte | Direct spectrophotometric method using KO_2_ | (42) |
| G94V | 54% | 1 family vs controls | - | - | Erythrocyte | Direct spectrophotometric method using KO_2_ | (42) |
| E101G | 32% | 1 patients vs 25 controls | 44 | >48 | Erythrocyte | Epinephrine autoxidation assay | (26) |
| E101G | 64% | 2 families vs controls | - | - | Erythrocyte | Direct spectrophotometric method using KO_2_ | (42) |
| S108Lfs*15 | 48% | 1 patient vs 170 controls | 64 | >84 | Erythrocyte | Direct spectrophotometric method using KO_2_ | (43) |
| D110Y | 98% | 1 patient vs 5 control | 51 | 72 | Erythrocyte | (44) | (45) |
| I113T | 42% | 3 patients vs 25 controls | 44 | >11 | Erythrocyte | Epinephrine autoxidation assay | (26) |
| I114T | 53% | 2 patients vs 25 controls | 61 | >19 | Erythrocyte | Epinephrine autoxidation assay | (26) |
| I114T | 51% | 3 families vs controls | - | - | Erythrocyte | Direct spectrophotometric method using KO_2_ | (42) |
| I114T | 81% | 1 patient vs 28 controls | 68 (age at death) | - | Brain area 40 | Epinephrine autoxidation assay | (27) |
| I114T | 61% | 1 patient vs 16 controls | 68 (age at death) | - | Cerebellum | Epinephrine autoxidation assay | (27) |
| L118V | 88% | 1 patient vs 4 controls | 36 | >116 | Erythrocytes | - | (28) |
| L118V | 92% | 4 patients vs 7 controls | 40 | >96 | Erythrocytes | Direct spectrophotometric method using KO_2_ | (46) |
| D125Tfs*24 | 47% | 1 patient vs 4 controls | 55 | >107 | Erythrocyte | - | (28) |
| D126H | 53% | 1 family vs controls | - | - | Erythrocyte | Direct spectrophotometric method using KO_2_ | (42) |
| L127Gfs*6 | 30% | 1 patient vs 10 controls | 42 | 24 | Erythrocyte | xanthine oxidation assay | (47) |
| L127Gfs*6 | 42% | 1 patient vs 5 controls | 59 | 32 | Frontal lobe | Direct spectrophotometric method using KO_2_ | (48) |
| L127Gfs*6 | 47% | 1 patient vs 5 controls | 59 | 32 | Temporal lobe | Direct spectrophotometric method using KO_2_ | (48) |
| L127Gfs*6 | 51% | 1 patient vs 5 controls | 59 | 32 | Precentral gyrus | Direct spectrophotometric method using KO_2_ | (48) |
| L127Gfs*6 | 60% | 1 patient vs 5 controls | 59 | 32 | Cervical ventral horn | Direct spectrophotometric method using KO_2_ | (48) |
| L127Gfs*6 | 46% | 1 patient vs 5 controls | 59 | 32 | Cervical dorsal horn | Direct spectrophotometric method using KO_2_ | (48) |
| L127Gfs*6 | 43% | 1 patient vs 5 controls | 59 | 32 | Cervical dorsal column | Direct spectrophotometric method using KO_2_ | (48) |
| L127Gfs*6 | 59% | 1 patient vs 5 controls | 59 | 32 | Cervical corticospinal tract | Direct spectrophotometric method using KO_2_ | (48) |
| L127Gfs*6 | 63% | 1 patient vs 5 controls | 59 | 32 | Lumbar ventral horn | Direct spectrophotometric method using KO_2_ | (48) |
| L127Gfs*6 | 62% | 1 patient vs 5 controls | 59 | 32 | Lumbar dorsal horn | Direct spectrophotometric method using KO_2_ | (48) |
| L127Gfs*6 | 57% | 1 patient vs 5 controls | 59 | 32 | Lumbar dorsal column | Direct spectrophotometric method using KO_2_ | (48) |
| L127Gfs*6 | 53% | 1 patient vs 5 controls | 59 | 32 | Lumbar corticospinal tract | Direct spectrophotometric method using KO_2_ | (48) |
| L127S | 72% | 3 patients vs 16 controls | 59 | 32 | Erythrocyte | Xanthine oxidation assay | (49) |
| N132Qfs*5 | 58.1% | 1 patient vs families without mutation | 33 | na | Erythrocyte | SOD-typed assay kit | (50) |
| E133insTT | 46% | 1 family vs controls | - | - | Erythrocyte | Direct spectrophotometric method using KO_2_ | (42) |
| G139V | 46% | 1 patient vs control | 34 | 20 | Erythrocyte | - | (51) |
| N140H | 61% | 1 patient vs 2 families without mutation | 59 | 20 | Erythrocyte | RANSOD SD125 kit | (52) |
| L145F | 67% | 3 patients vs 12 controls | 57 | 59 | Erythrocyte | Xanthine oxidation assay | (53) |
| L145F | 67% | 3 patients vs 12 controls | 57 | 59 | Mononuclear cells | Xanthine oxidation assay | (53) |
| L145F | 77% | 8 patients vs 31 controls | - | - | Erythrocyte | Direct spectrophotometric method using KO_2_ | (54) |
| I150T | 53% | 1 family vs controls | - | - | Erythrocyte | Direct spectrophotometric method using KO_2_ | (42) |
| c.240-7T>G | 46% | 1 patient vs 4 controls | 42 | 20 | Erythrocyte | - | (28) |
| c.240-7T>G | 44% | 1 patient vs 2 healthy controls and 1 SBMA control | 42 | 20 | Erythrocyte | Direct spectrophotometric method using KO_2_ | (55) |
| c.240-7T>G | 71% | 1 patient vs 1 SBMA control | 42 | 20 | Fibroblast | Direct spectrophotometric method using KO_2_ | (55) |

Table S5. Protein level of mutant SOD1 in human tissues

| Variant | Protein level | Compartments | Tissue | Method | Ref |
| --- | --- | --- | --- | --- | --- |
| A5V | 80% | 2 patients vs 4 controls | Anterior horn cells | Immunustaining | (25) |
| A5V | 51% | 4 patients vs 25 controls | Erythrocyte | ELISA | (26) |
| A5S | 56% | Mutant/normal SOD1 | Erythrocyte | LC-ESIMS | (56) |
| C7S | 78% | 5 carriers vs 5 controls | Erythrocyte | WB | (30) |
| V15G | 33% | Mutant/normal SOD1 | Erythrocyte | LC-ESIMS | (56) |
| G38R | 56% | Mutant/normal SOD1 | Erythrocyte | LC-ESIMS | (56) |
| H47R | 15% | Mutant/normal SOD1 | Erythrocyte | LC-ESIMS | (56) |
| H47R | 43% | Mutant/normal SOD1 | Spinal cord | LC-ESIMS | (57) |
| H47R | 47% | Mutant/normal SOD1 | Iliopsoas muscle | LC-ESIMS | (57) |
| H47R | 60% | Mutant/normal SOD1 | Diaphragm | LC-ESIMS | (57) |
| H47R | 14% | Mutant/normal SOD1 | Erythrocyte | LC-ESIMS | (57) |
| D77Y | 61% | Mutant/normal SOD1 | Erythrocyte | LC-ESIMS | (56) |
| L85F | 82% | 4 patients vs 4 controls | Erythrocyte | ELISA | (36) |
| L85F | 86% | 4 patients vs 11 controls | Lymphocyte | ELISA | (36) |
| L85F | 73% | 3 patients vs 11 controls | Erythrocyte | ELISA | (37) |
| N87S | 12% | Mutant/normal SOD1 | Erythrocyte | LC-ESIMS | (56) |
| A90V | 54% | Mutant/normal SOD1 | Erythrocyte | LC-ESIMS | (56) |
| G94D | 51% | 6 patients vs 25 controls | Erythrocyte | ELISA | (26) |
| G94A | 70% | 1 patients vs 25 controls | Erythrocyte | ELISA | (26) |
| E101G | 27% | 1 patients vs 25 controls | Erythrocyte | ELISA | (26) |
| I113T | 44% | 3 patients vs 25 controls | Erythrocyte | ELISA | (26) |
| I114T | 49% | 2 patients vs 25 controls | Erythrocyte | ELISA | (26) |
| I114T | 29% | Mutant/normal SOD1 | Erythrocyte | LC-ESIMS | (56) |
| I114F | 23% | Mutant/normal SOD1 | Erythrocyte | LC-ESIMS | (56) |
| L127S | 67% | 1 patient vs 14 controls | CSF | ELISA | (49) |
| L127S | 50% | Mutant/normal SOD1 | Erythrocyte | LC-ESIMS | (56) |
| G142E | 28% | Mutant/normal SOD1 | Erythrocyte | LC-ESIMS | (56) |
| L145F | 29% | Mutant/normal SOD1 | Erythrocyte | LC-ESIMS | (56) |

Table S6. Half-lives of mutant SOD1 proteins

| Variant | Half-life | Compartments | Species | Tissue | Major Method | Ref |
| --- | --- | --- | --- | --- | --- | --- |
| A5V | 7.5 h (25%) | Wild type (30 h) | Monkey | Transient transfected COS-1 cells | Radiolabel  immunoblot | (32) |
| A5V | 4 h (soluble, 24%)  7 h (with aggregates, 41%) | WT (17 h) | Mouse | NSC-34 | Dendra2 + microscopy | (58) |
| A5V | 12.4 d (43%) | WT (29 d) | Human | CSF | Stable isotope labeling  kinetics | (59) |
| A5T | 18 h (23%) | Wild type (78 h) | Monkey | Transient transfected COS-7 cells | Radiolabel  immunoblot | (60) |
| G38R | 13 h (43%) | Wild type (30 h) | Monkey | Transient transfected COS-1 cells | Radiolabel  immunoblot | (32) |
| G42D | 10 h (33%) | Wild type (30 h) | Monkey | Transient transfected COS-1 cells | Radiolabel  immunoblot | (32) |
| H47R | >24 h | Wild type | Monkey | Transient transfected COS-1 cells | Radiolabel  immunoblot | (61) |
| H49Q | >24 h | Wild type | Monkey | Transient transfected COS-1 cells | Radiolabel  immunoblot | (61) |
| G86R | 7.5 h (25%) | Wild type (30 h) | Monkey | Transient transfected COS-1 cells | Radiolabel  immunoblot | (32) |
| G94A | 7.8 d (71%) | Wild type (11 d) | Rat | Whole body | Stable isotope-labeling + mass spectrometric detection | (62) |
| G94A | 1.4 d (82%) | Wild type (1.7 d) | Rat | Liver | Stable isotope-labeling + mass spectrometric detection | (62) |
| G94A | 3.7 d (109%) | Wild type (3.4 d) | Rat | Kidney | Stable isotope-labeling + mass spectrometric detection | (62) |
| G94A | 6.7 d (72%) | Wild type (9.3 d) | Rat | Cortex | Stable isotope-labeling + mass spectrometric detection | (62) |
| G94A | 9.4 d (63%) | Wild type (14.9 d) | Rat | CSF | Stable isotope-labeling + mass spectrometric detection | (62) |
| G94A | 9.0 d (57%) | Wild type (15.9 d) | Rat | Spinal cord | Stable isotope-labeling + mass spectrometric detection | (62) |
| G94C | 16 h (53%) | Wild type (30 h) | Monkey | Transient transfected COS-1 cells | Radiolabel  immunoblot | (32) |
| I114T | 20 h (67%) | Wild type (30 h) | Monkey | Transient transfected COS-1 cells | Radiolabel  immunoblot | (32) |

Table S7. Histological analysis of aggregations

| Variant | Description | Species | Ref |
| --- | --- | --- | --- |
| A5V | Anterior horn neuron of posterior column, irregular intracellular hyaline inclusions with eosinophilic inner portion and a surrounding pale zone in eosin staining, SOD1/Ubiquitin/pNF/ HtrA2/Omi+ | Human | (63-65) |
| A5V | Lumbar motor neuron, intracellular large skein-like inclusions, SOD1+ | Human | (66) |
| G28delGP_hom | Motor neuron, hyaline conglomerate floccular inclusions in hematoxylin staining, pNF/misfolded SOD1+ | Human | (31) |
| G38R | Anterior horn of sacral spinal cord, hyaline inclusion in HE staining (few) | Human | (67) |
| G73C | 1. Lower motor neuron, intracellular inclusions in HE staining, SOD1+  2. Lower motor neuron, in axons and perikaryon, ubiquitin+  3. Lower motor neuron, cytoplasmic aggregate of short fuzzy filaments, SOD1+ | Human | (68) |
| G86R | 1. Astrocyte, SOD1/ubiquitin+ inclusions  2. Motor neuron, SOD1/ubiquitin+ aggregations | Mouse | (69) |
| G86R | 1. Ventral portion of spinal cord, hyaline inclusion in HE staining, SOD1+  2. Ventral portion of spinal cord, not identifiable with HE staining, uniform SOD1+ aggregates | Mouse | (70) |
| D91A_hom | 1. Motor neuron, small granular inclusions, misfolded SOD1+  2. Motor neurons in brain stem, large cytoplasmic inclusions, p62+  3. Substantia nigra neurons, intranuclear, Marinesco bodies | Human | (66) |
| G94A | 1. Ventral portion of spinal cord, hyaline inclusion in HE staining, SOD1+  2. Ventral portion of spinal cord, not identifiable with HE staining, uniform SOD1+ aggregates | Mouse | (70) |
| G94S | No Lewy body-like hyaline inclusions (LBHI) nor SOD1/ubiquitin positive aggregations | Human | (71) |
| C112Y | Motor neuron, hyaline inclusions, SOD1+, TDP/ubiquitin+ (halo portion) | Human | (72) |
| I113T | Neuronal cell bodies and neuronal processes, LBHI with eosinophilic cores and pale halos in HE staining, HtrA2/Omi+ | Human | (63) |
| I114T | Spinal motor neurons, intracellular conglomerate inclusions of pale eosinophilic and multifocal large inclusions in HE staining, HtrA2/Omi+ | Human | (63) |
| L127Gfs*6 | Lumbar spinal cord motor neurons, intracellular LBHI, HtrA2/Omi+ | Human | (63) |
| L127Gfs*6 | 1. Spinal cord motor neurons, small inclusions, ubiquitin/SOD1mut+  2. Spinal cord motor neurons, skein-like inclusion, ubiquitin/SOD1mut+  3. Spinal cord motor neurons, LBHI, SOD1mut+  4. Spinal cord astrocytes, an inclusion, SOD1mut+ | Human | (48) |
| G139V | 1. Neurons in lumbar anterior horn of spinal cord, intracellular inclusions, ubiquitin+  2. Neurons in precentral region of motor cortex, intracellular inclusions, ubiquitin/SOD1+ | Human | (51) |
| G142* | 1. Anterior horn cells of the spinal cord, skein-like inclusions, SOD1/p62+  2. Neurons in hypoglossal nucleus, conglomerate inclusions of pale eosinophilic in HE staining, SOD1/pNF+  3. Neurons in frontal cortex, many round inclusions slightly eosinophilic in HE staining, α-internexin/SOD1+  4. Neurons in frontal cortex, tangle-like cytoplasmic inclusions, pNF/SOD1/α-internexin+  5. Neurons in frontal cortex, conglomerate inclusions, pNF/SOD1/α-internexin+ | Human | (73) |

1. Cudkowicz ME, McKenna-Yasek D, Sapp PE, Chin W, Geller B, Hayden DL, Schoenfeld DA, Hosler BA, Horvitz HR, Brown RH. Epidemiology of mutations in superoxide dismutase in amyotrophic lateral sclerosis. Ann Neurol. 1997 Feb;41(2):210-21.

2. Battistini S, Giannini F, Greco G, Bibbo G, Ferrera L, Marini V, Causarano R, Casula M, Lando G, Patrosso MC, Caponnetto C, Origone P, Marocchi A, Del Corona A, Siciliano G, Carrera P, Mascia V, Giagheddu M, Carcassi C, Orru S, Garre C, Penco S. SOD1 mutations in amyotrophic lateral sclerosis. Results from a multicenter Italian study. J Neurol. 2005 Jul;252(7):782-8.

3. Rabe M, Felbecker A, Waibel S, Steinbach P, Winter P, Muller U, Ludolph AC. The epidemiology of CuZn-SOD mutations in Germany: a study of 217 families. J Neurol. 2010 Aug;257(8):1298-302.

4. Lysogorskaia EV, Abramycheva NY, Zakharova MN, Stepanova MS, Moroz AA, Rossokhin AV, Illarioshkin SN. Genetic studies of Russian patients with amyotrophic lateral sclerosis. Amyotroph Lateral Scler Frontotemporal Degener. 2015;17(1-2):135-41.

5. Cirulli ET, Lasseigne BN, Petrovski S, Sapp PC, Dion PA, Leblond CS, Couthouis J, Lu YF, Wang Q, Krueger BJ, Ren Z, Keebler J, Han Y, Levy SE, Boone BE, Wimbish JR, Waite LL, Jones AL, Carulli JP, Day-Williams AG, Staropoli JF, Xin WW, Chesi A, Raphael AR, McKenna-Yasek D, Cady J, Vianney de Jong JM, Kenna KP, Smith BN, Topp S, Miller J, Gkazi A, Consortium FS, Al-Chalabi A, van den Berg LH, Veldink J, Silani V, Ticozzi N, Shaw CE, Baloh RH, Appel S, Simpson E, Lagier-Tourenne C, Pulst SM, Gibson S, Trojanowski JQ, Elman L, McCluskey L, Grossman M, Shneider NA, Chung WK, Ravits JM, Glass JD, Sims KB, Van Deerlin VM, Maniatis T, Hayes SD, Ordureau A, Swarup S, Landers J, Baas F, Allen AS, Bedlack RS, Harper JW, Gitler AD, Rouleau GA, Brown R, Harms MB, Cooper GM, Harris T, Myers RM, Goldstein DB. Exome sequencing in amyotrophic lateral sclerosis identifies risk genes and pathways. Science. 2015 Mar 27;347(6229):1436-41.

6. Hou L, Jiao B, Xiao T, Zhou L, Zhou Z, Du J, Yan X, Wang J, Tang B, Shen L. Screening of SOD1, FUS and TARDBP genes in patients with amyotrophic lateral sclerosis in central-southern China. Sci Rep. 2016 Sep 8;6:32478.

7. Nakamura R, Sone J, Atsuta N, Tohnai G, Watanabe H, Yokoi D, Nakatochi M, Watanabe H, Ito M, Senda J, Katsuno M, Tanaka F, Li Y, Izumi Y, Morita M, Taniguchi A, Kano O, Oda M, Kuwabara S, Abe K, Aiba I, Okamoto K, Mizoguchi K, Hasegawa K, Aoki M, Hattori N, Tsuji S, Nakashima K, Kaji R, Sobue G, Japanese Consortium for Amyotrophic Lateral Sclerosis R. Next-generation sequencing of 28 ALS-related genes in a Japanese ALS cohort. Neurobiol Aging. 2016 Mar;39:219 e1-8.

8. Wei Q, Zhou Q, Chen Y, Ou R, Cao B, Xu Y, Yang J, Shang HF. Analysis of SOD1 mutations in a Chinese population with amyotrophic lateral sclerosis: a case-control study and literature review. Sci Rep. 2017 Mar 14;7:44606.

9. Muller K, Brenner D, Weydt P, Meyer T, Grehl T, Petri S, Grosskreutz J, Schuster J, Volk AE, Borck G, Kubisch C, Klopstock T, Zeller D, Jablonka S, Sendtner M, Klebe S, Knehr A, Gunther K, Weis J, Claeys KG, Schrank B, Sperfeld AD, Hubers A, Otto M, Dorst J, Meitinger T, Strom TM, Andersen PM, Ludolph AC, Weishaupt JH, German ALSnMNDNET. Comprehensive analysis of the mutation spectrum in 301 German ALS families. J Neurol Neurosurg Psychiatry. 2018 Aug;89(8):817-27.

10. Liu ZJ, Lin HX, Wei Q, Zhang QJ, Chen CX, Tao QQ, Liu GL, Ni W, Gitler AD, Li HF, Wu ZY. Genetic Spectrum and Variability in Chinese Patients with Amyotrophic Lateral Sclerosis. Aging Dis. 2019 Dec;10(6):1199-206.

11. Tang L, Ma Y, Liu XL, Chen L, Fan DS. Better survival in female SOD1-mutant patients with ALS: a study of SOD1-related natural history. Transl Neurodegener. 2019;8:2.

12. Farhan SMK, Howrigan DP, Abbott LE, Klim JR, Topp SD, Byrnes AE, Churchhouse C, Phatnani H, Smith BN, Rampersaud E, Wu G, Wuu J, Shatunov A, Iacoangeli A, Al Khleifat A, Mordes DA, Ghosh S, Consortium A, Consortium F, Project Min EC, Consortium CR, Eggan K, Rademakers R, McCauley JL, Schule R, Zuchner S, Benatar M, Taylor JP, Nalls M, Gotkine M, Shaw PJ, Morrison KE, Al-Chalabi A, Traynor B, Shaw CE, Goldstein DB, Harms MB, Daly MJ, Neale BM. Exome sequencing in amyotrophic lateral sclerosis implicates a novel gene, DNAJC7, encoding a heat-shock protein. Nat Neurosci. 2019 Dec;22(12):1966-74.

13. Chen W, Xie Y, Zheng M, Lin J, Huang P, Pei Z, Yao X. Clinical and genetic features of patients with amyotrophic lateral sclerosis in southern China. Eur J Neurol. 2020 Jun;27(6):1017-22.

14. Chio A, Mora G, Moglia C, Manera U, Canosa A, Cammarosano S, Ilardi A, Bertuzzo D, Bersano E, Cugnasco P, Grassano M, Pisano F, Mazzini L, Calvo A, Piemonte, Valle d'Aosta Register for ALS. Secular Trends of Amyotrophic Lateral Sclerosis: The Piemonte and Valle d'Aosta Register. JAMA Neurol. 2017 Sep 1;74(9):1097-104.

15. Chio A, Moglia C, Canosa A, Manera U, D'Ovidio F, Vasta R, Grassano M, Brunetti M, Barberis M, Corrado L, D'Alfonso S, Iazzolino B, Peotta L, Sarnelli MF, Solara V, Zucchetti JP, De Marchi F, Mazzini L, Mora G, Calvo A. ALS phenotype is influenced by age, sex, and genetics: A population-based study. Neurology. 2020 Feb 25;94(8):e802-e10.

16. Nunes Goncalves JP, Leoni TB, Martins MP, Peluzzo TM, Dourado MET, Jr., Saute JAM, Paranhos Miranda Covaleski AP, Bulle de Oliveira AS, Claudino R, Marques W, Jr., Nucci A, Franca MC, Jr. Genetic epidemiology of familial ALS in Brazil. Neurobiol Aging. 2021 Jun;102:227 e1- e4.

17. Chen LX, Xu HF, Wang PS, Yang XX, Wu ZY, Li HF. SOD1 Mutation Spectrum and Natural History of ALS Patients in a 15-Year Cohort in Southeastern China. Front Genet. 2021;12:746060.

18. Bartoletti-Stella A, Vacchiano V, De Pasqua S, Mengozzi G, De Biase D, Bartolomei I, Avoni P, Rizzo G, Parchi P, Donadio V, Chio A, Pession A, Oppi F, Salvi F, Liguori R, Capellari S, BoReAls. Targeted sequencing panels in Italian ALS patients support different etiologies in the ALS/FTD continuum. J Neurol. 2021 Oct;268(10):3766-76.

19. Liu Z, Yuan Y, Wang M, Ni J, Li W, Huang L, Hu Y, Liu P, Hou X, Hou X, Du J, Weng L, Zhang R, Niu Q, Tang J, Jiang H, Shen L, Tang B, Wang J. Mutation spectrum of amyotrophic lateral sclerosis in Central South China. Neurobiol Aging. 2021 Nov;107:181-8.

20. Nel M, Mahungu AC, Monnakgotla N, Botha GR, Mulder NJ, Wu G, Rampersaud E, van Blitterswijk M, Wuu J, Cooley A, Myers J, Rademakers R, Taylor JP, Benatar M, Heckmann JM. Revealing the Mutational Spectrum in Southern Africans With Amyotrophic Lateral Sclerosis. Neurol Genet. 2022 Feb;8(1):e654.

21. Olsen CG, Busk OL, Aanjesen TN, Alstadhaug KB, Bjorna IK, Braathen GJ, Breivik KL, Demic N, Flemmen HO, Hallerstig E, HogenEsch I, Holla OL, Jontvedt AB, Kampman MT, Kleveland G, Kvernmo HB, Ljostad U, Maniaol A, Morsund AH, Nakken O, Novy C, Rekand T, Schluter K, Schuler S, Tveten K, Tysnes OB, Holmoy T, Hoyer H. Genetic Epidemiology of Amyotrophic Lateral Sclerosis in Norway: A 2-Year Population-Based Study. Neuroepidemiology. 2022;56(4):271-82.

22. Berdynski M, Miszta P, Safranow K, Andersen PM, Morita M, Filipek S, Zekanowski C, Kuzma-Kozakiewicz M. SOD1 mutations associated with amyotrophic lateral sclerosis analysis of variant severity. Sci Rep. 2022 Jan 7;12(1):103.

23. Chen YP, Yu SH, Wei QQ, Cao B, Gu XJ, Chen XP, Song W, Zhao B, Wu Y, Sun MM, Liu FF, Hou YB, Ou RW, Zhang LY, Liu KC, Lin JY, Xu XR, Li CY, Yang J, Jiang Z, Liu J, Cheng YF, Xiao Y, Chen K, Feng F, Cai YY, Li SR, Hu T, Yuan XQ, Guo XY, Liu H, Han Q, Zhou QQ, Shao N, Li JP, Pan PL, Ma S, Shang HF. Role of genetics in amyotrophic lateral sclerosis: a large cohort study in Chinese mainland population. J Med Genet. 2022 Sep;59(9):840-9.

24. Vazquez-Costa JF, Borrego-Hernandez D, Paradas C, Gomez-Caravaca MT, Rojas-Garcia R, Varona L, Povedano M, Garcia-Sobrino T, Jerico Pascual I, Gutierrez A, Riancho J, Turon-Sans J, Assialioui A, Perez-Tur J, Sevilla T, Esteban Perez J, Garcia-Redondo A, Alsgesco. Characterizing SOD1 mutations in Spain. The impact of genotype, age, and sex in the natural history of the disease. Eur J Neurol. 2022 Dec 9.

25. Rosen DR, Bowling AC, Patterson D, Usdin TB, Sapp P, Mezey E, McKenna-Yasek D, O’Regan J, Rahmani Z, Ferrante RJ, Brownstein MJ, Kowall NW, Beal MF, Horvitz HR, Brown JRH. A frequent ala 4 to val superoxide dismutase-1 mutation is associated with a rapidly progressive familial amyotrophic lateral sclerosis. Human Molecular Genetics. 1994;3.

26. Bowling AC, Barkowski EE, McKenna-Yasek D, Sapp P, Horvitz HR, Beal MF, Brown RH, Jr. Superoxide dismutase concentration and activity in familial amyotrophic lateral sclerosis. Journal of neurochemistry. 1995 May;64(5):2366-9.

27. Browne SE, Bowling AC, Baik MJ, Gurney M, Brown RH, Jr., Beal MF. Metabolic dysfunction in familial, but not sporadic, amyotrophic lateral sclerosis. Journal of neurochemistry. 1998 Jul;71(1):281-7.

28. Keskin I, Forsgren E, Lange DJ, Weber M, Birve A, Synofzik M, Gilthorpe JD, Andersen PM, Marklund SL. Effects of Cellular Pathway Disturbances on Misfolded Superoxide Dismutase-1 in Fibroblasts Derived from ALS Patients. PLoS One. 2016;11(2):e0150133.

29. Marklund S. Spectrophotometric study of spontaneous disproportionation of superoxide anion radical and sensitive direct assay for superoxide dismutase. Journal of Biological Chemistry. 1976;251(23):7504-7.

30. Brotherton T, Polak M, Kelly C, Birve A, Andersen P, Marklund SL, Glass JD. A novel ALS SOD1 C6S mutation with implications for aggregation related toxicity and genetic counseling. Amyotroph Lateral Scler. 2011 May;12(3):215-9.

31. Zinman L, Liu HN, Sato C, Wakutani Y, Marvelle AF, Moreno D, Morrison KE, Mohlke KL, Bilbao J, Robertson J, Rogaeva E. A mechanism for low penetrance in an ALS family with a novel SOD1 deletion. Neurology. 2009 Mar 31;72(13):1153-9.

32. Borchelt DR, Lee MK, Slunt HS, Guarnieri M, Xu ZS, Wong PC, Brown RH, Jr., Price DL, Sisodia SS, Cleveland DW. Superoxide dismutase 1 with mutations linked to familial amyotrophic lateral sclerosis possesses significant activity. Proc Natl Acad Sci U S A. 1994 Aug 16;91(17):8292-6.

33. Robberecht W, Sapp P, Viaene MK, Rosen D, McKenna-Yasek D, Haines J, Horvitz R, Theys P, Brown R, Jr. Cu/Zn superoxide dismutase activity in familial and sporadic amyotrophic lateral sclerosis. Journal of neurochemistry. 1994 Jan;62(1):384-7.

34. Tsuda T, Munthasser S, Fraser PE, Percy ME, Rainero I, Vaula G, Pinessi L, Bergamini L, Vignocchi G, McLachlan DR, et al. Analysis of the functional effects of a mutation in SOD1 associated with familial amyotrophic lateral sclerosis. Neuron. 1994 Sep;13(3):727-36.

35. Fahmy N, Muller K, Andersen PM, Marklund SL, Otto M, Ludolph AC, Hamdi N. A novel homozygous p.Ser69Pro SOD1 mutation causes severe young-onset ALS with decreased enzyme activity. J Neurol. 2023 Mar;270(3):1770-3.

36. Ceroni M, Malaspina A, Poloni TE, Alimonti D, Rognoni F, Habgood J, Imbesi F, Antonelli P, Alfonsi E, Curti D, deBelleroche J. Clustering of ALS patients in central Italy due to the occurrence of the L84F SOD1 gene mutation. Neurology. 1999;53.

37. Curti D, Alimonti D, Malaspina A, Feletti F, Tessera S, Finotti N, Rehak L, Mazzini L, Zerbi F, Poloni TE, Ceroni M. SOD1 activity and protective factors in familial ALS patients with L84F SOD1 mutation. ALS and other motor neuron disorders. 2002;3.

38. Orrell RW, Marklund SL, deBelleroche JS. Familial ALS is associated with mutations in all exons of SOD1: a novel mutation in exon 3 (Gly72Ser). Journal of Neurological Sciences. 1997;153.

39. Andersen PM, Nilsson P, Forsgren L, Marklund SL. CuZn-superoxide dismutase, extracellular superoxide dismutase, and glutathione peroxidase in blood from individuals homozygous for Asp90Ala CuZu-superoxide dismutase mutation. Journal of neurochemistry. 1998 Feb;70(2):715-20.

40. Morita M, Abe K, Takahashi M, Onodera Y, Okumura H, Niino M, Tashiro K, Nakano I, Itoyama Y. A novel mutation Asp90Val in the SOD1 gene associated with Japanese familial ALS. Eur J Neurol. 1998;5.

41. Kawata A, Kato S, Hayashi H, Hirai S. Prominent sensory and autonomic disturbances in familial amyotrophic lateral sclerosis with a Gly93Ser mutation in the SOD1 gene. J Neurol Sci. 1997 Dec 9;153(1):82-5.

42. Orrell RW, Habgood JJ, Gardiner I, King AW, Bowe FA, Hallewell RA, Marklund SL, Greenwood J, Lane RJ, deBelleroche J. Clinical and functional investigation of 10 missense mutations and a novel frameshift insertion mutation of the gene for copper-zinc superoxide dismutase in UK families with amyotrophic lateral sclerosis. Neurology. 1997 Mar;48(3):746-51.

43. Canosa A, De Marco G, Lomartire A, Rinaudo MT, Di Cunto F, Turco E, Barberis M, Brunetti M, Casale F, Moglia C, Calvo A, Marklund SL, Andersen PM, Mora G, Chio A. A novel p.Ser108LeufsTer15 SOD1 mutation leading to the formation of a premature stop codon in an apparently sporadic ALS patient: insights into the underlying pathomechanisms. Neurobiol Aging. 2018 Dec;72:189 e11- e17.

44. Gilles L, Ferradini C, Foos J, Pucheault J, Allard D, Sinet PM, Jerome H. The estimation of red cell superoxide dismutase activity by pulse radiolysis in normal and trisomic 21 subjects. FEBS Lett. 1976 Oct 15;69(1):55-8.

45. Naini A, Mehrazin M, Lu J, Gordon P, Mitsumoto H. Identification of a novel D109Y mutation in Cu/Zn superoxide dismutase (sod1) gene associated with amyotrophic lateral sclerosis. J Neurol Sci. 2007 Mar 15;254(1-2):17-21.

46. Synofzik M, Ronchi D, Keskin I, Basak AN, Wilhelm C, Gobbi C, Birve A, Biskup S, Zecca C, Fernandez-Santiago R, Kaugesaar T, Schols L, Marklund SL, Andersen PM. Mutant superoxide dismutase-1 indistinguishable from wild-type causes ALS. Hum Mol Genet. 2012 Aug 15;21(16):3568-74.

47. Kadekawa J, Fujimura H, Ogawa Y, Hattori N, Kaido M, Nishimura T, Yoshikawa H, Shirahata N, Sakoda S, Yanagihara T. A clinicopathological study of a patient with familial amyotrophic lateral sclerosis associated with a two base pair deletion in the copper/zinc superoxide dismutase (SOD1) gene. Acta Neuropathol. 1997 Dec;94(6):617-22.

48. Jonsson PA, Ernhill K, Andersen PM, Bergemalm D, Brannstrom T, Gredal O, Nilsson P, Marklund SL. Minute quantities of misfolded mutant superoxide dismutase-1 cause amyotrophic lateral sclerosis. Brain. 2004 Jan;127(Pt 1):73-88.

49. Ihara Y, Nobukuni K, Takata H, Hayabara T. Oxidative stress and metal content in blood and cerebrospinal fluid of amyotrophic lateral sclerosis patients with and without a Cu, Zn-superoxide dismutase mutation. Neurological Research. 2005;27.

50. Chen S, Li M, Zhu W, Mao F, Wang J, Sun Z, Huang X. A novel 10-base pair insertion mutation in exon 5 of the SOD1 gene in a Chinese family with amyotrophic lateral sclerosis. Neurobiol Aging. 2016 Sep;45:212 e1- e4.

51. Masrori P, Ospitalieri S, Forsberg K, Moens TG, Poesen K, Race V, Brannstrom T, Andersen PM, Thal DR, Van Damme P. Respiratory onset of amyotrophic lateral sclerosis in a pregnant woman with a novel SOD1 mutation. Eur J Neurol. 2022 Apr;29(4):1279-83.

52. Nogales-Gadea G, Garcia-Arumi E, Andreu AL, Cervera C, Gamez J. A novel exon 5 mutation (N139H) in the SOD1 gene in a Spanish family associated with incomplete penetrance. J Neurol Sci. 2004 Apr 15;219(1-2):1-6.

53. Mase G, Ros S, Gemma A, Bonfigli L, Carraro N, Cazzato G, Rolfo M, Zanconati F, Sepcic J, Jurjevic A, Pirulli D, Boniotto M, Zezlina S, Crovella S, Amoroso A. ALS with variable phenotypes in a six-generation family caused by leu144phe mutation in the SOD1 gene. J Neurol Sci. 2001 Oct 15;191(1-2):11-8.

54. Nikolic-Kokic A, Stevic Z, Blagojevic D, Davidovic B, Jones DR, Spasic MB. Alterations in anti-oxidative defence enzymes in erythrocytes from sporadic amyotrophic lateral sclerosis (SALS) and familial ALS patients. Clin Chem Lab Med. 2006;44(5):589-93.

55. Birve A, Neuwirth C, Weber M, Marklund SL, Nilsson AC, Jonsson PA, Andersen PM. A novel SOD1 splice site mutation associated with familial ALS revealed by SOD activity analysis. Hum Mol Genet. 2010 Nov 1;19(21):4201-6.

56. Sato T, Nakanishi T, Yamamoto Y, Andersen PM, Ogawa Y, Fukada K, Zhou Z, Aoike F, Sugai F, Nagano S, Hirata S, Ogawa M, Nakano R, Ohi T, Kato T, Nakagawa M, Hamasaki T, Shimizu A, Sakoda S. Rapid disease progression correlates with instability of mutant SOD1 in familial ALS. Neurology. 2005 Dec 27;65(12):1954-7.

57. Arisato T, Okubo R, Arata H, Abe K, Fukada K, Sakoda S, Shimizu A, Qin XH, Izumo S, Osame M, Nakagawa M. Clinical and pathological studies of familial amyotrophic lateral sclerosis (FALS) with SOD1 H46R mutation in large Japanese families. Acta Neuropathol. 2003 Dec;106(6):561-8.

58. Farrawell NE, Yerbury JJ. Mutant Cu/Zn Superoxide Dismutase (A4V) Turnover Is Altered in Cells Containing Inclusions. Front Mol Neurosci. 2021;14:771911.

59. Ly CV, Ireland MD, Self WK, Bollinger J, Jockel-Balsarotti J, Herzog H, Allred P, Miller L, Doyle M, Anez-Bruzual I, Trikamji B, Hyman T, Kung T, Nicholson K, Bucelli RC, Patterson BW, Bateman RJ, Miller TM. Protein kinetics of superoxide dismutase-1 in familial and sporadic amyotrophic lateral sclerosis. Ann Clin Transl Neurol. 2023 Jun;10(6):1012-24.

60. Nakano R, Inuzuka T, Kikugawa K, Takahashi H, Sakimura K, Fujii J, Taniguchi N, Tsuji S. Instability of mutant Cu/Zn superoxide dismutase (Ala4Thr) associated with familial amyotrophic lateral sclerosis. Neuroscience letters. 1996 Jun 21;211(2):129-31.

61. Ratovitski T, Corson LB, Strain J, Wong P, Cleveland DW, Culotta VC, Borchelt DR. Variation in the biochemical/biophysical properties of mutant superoxide dismutase 1 enzymes and the rate of disease progression in familial amyotrophic lateral sclerosis kindreds. Hum Mol Genet. 1999 Aug;8(8):1451-60.

62. Crisp MJ, Mawuenyega KG, Patterson BW, Reddy NC, Chott R, Self WK, Weihl CC, Jockel-Balsarotti J, Varadhachary AS, Bucelli RC, Yarasheski KE, Bateman RJ, Miller TM. In vivo kinetic approach reveals slow SOD1 turnover in the CNS. J Clin Invest. 2015 Jul 1;125(7):2772-80.

63. Kawamoto Y, Ito H, Kobayashi Y, Suzuki Y, Akiguchi I, Fujimura H, Sakoda S, Kusaka H, Hirano A, Takahashi R. HtrA2/Omi-immunoreactive intraneuronal inclusions in the anterior horn of patients with sporadic and Cu/Zn superoxide dismutase (SOD1) mutant amyotrophic lateral sclerosis. Neuropathol Appl Neurobiol. 2010 Jun;36(4):331-44.

64. Hirano A, Kurland LT, Sayre GP. Familial amyotrophic lateral sclerosis. A subgroup characterized by posterior and spinocerebellar tract involvement and hyaline inclusions in the anterior horn cells. Arch Neurol. 1967 Mar;16(3):232-43.

65. Shibata N, Hirano A, Kobayashi M, Siddique T, Deng HX, Hung WY, Kato T, Asayama K. Intense superoxide dismutase-1 immunoreactivity in intracytoplasmic hyaline inclusions of familial amyotrophic lateral sclerosis with posterior column involvement. J Neuropathol Exp Neurol. 1996 Apr;55(4):481-90.

66. Forsberg KM, Graffmo KS, Stenvall E, Tabikh N, Marklund SL, Brannstrom T, Andersen PM. Widespread CNS pathology in amyotrophic lateral sclerosis homozygous for the D90A SOD1 mutation. Acta Neuropathol. 2023 Jan;145(1):13-28.

67. Inoue K, Fujimura H, Ogawa Y, Satoh T, Shimada K, Sakoda S. Familial amyotrophic lateral sclerosis with a point mutation (G37R) of the superoxide dismutase 1 gene: a clinicopathological study. Amyotroph Lateral Scler Other Motor Neuron Disord. 2002 Dec;3(4):244-7.

68. Stewart HG, Mackenzie IR, Eisen A, Brannstrom T, Marklund SL, Andersen PM. Clinicopathological phenotype of ALS with a novel G72C SOD1 gene mutation mimicking a myopathy. Muscle Nerve. 2006 May;33(5):701-6.

69. Bruijn LI, Becher MW, Anderson KL, Jenkins NA, Copeland NG, Sisodia SS, Rothstein JD, Borchelt DR, Price DL, Cleveland DW. ALS-linked SOD1 mutant G85R mediates damage to astrocytes and promotes rapidly progressive disease with SOD1-containing inclusions. Neuron. 1997;18.

70. Bruijn LI, Houseweart MK, Kato S, Anderson KL, Anderson SD, Ohama E, Reaume AG, Scott RW, Cleveland DW. Aggregation and motor neuron toxicity of an ALS-linked SOD1 mutant independent from wild-type SOD1. Science. 1998 Sep 18;281(5384):1851-4.

71. Suzuki M, Irie T, Watanabe T, Mikami H, Yamazaki T, Oyanagi K, Ono S. Familial amyotrophic lateral sclerosis with Gly93Ser mutation in Cu/Zn superoxide dismutase: a clinical and neuropathological study. J Neurol Sci. 2008 May 15;268(1-2):140-4.

72. Sumi H, Kato S, Mochimaru Y, Fujimura H, Etoh M, Sakoda S. Nuclear TAR DNA binding protein 43 expression in spinal cord neurons correlates with the clinical course in amyotrophic lateral sclerosis. J Neuropathol Exp Neurol. 2009 Jan;68(1):37-47.

73. Nakamura M, Bieniek KF, Lin WL, Graff-Radford NR, Murray ME, Castanedes-Casey M, Desaro P, Baker MC, Rutherford NJ, Robertson J, Rademakers R, Dickson DW, Boylan KB. A truncating SOD1 mutation, p.Gly141X, is associated with clinical and pathologic heterogeneity, including frontotemporal lobar degeneration. Acta Neuropathol. 2015 Jul;130(1):145-57.
